# Peripheral blood microarray-based transcriptomic and epigenetic analyses identify immune, inflammation, and metabolic dysregulation in Alzheimer’s disease

**DOI:** 10.1038/s44400-026-00100-z

**Published:** 2026-08-31

**Authors:** Brendan A. Mitchell, Isabella Hausle, Sara Smith, Pamela Thropp, Marina Sirota, Duygu Tosun

**Affiliations:** 1 Department of Radiology and Biomedical Imaging, University of California, San Francisco, CA USA; 2 UC Berkeley - UCSF Graduate Program in Bioengineering, Berkeley, CA USA; 3 Department of Medicine, Division of Rheumatology, University of California, San Francisco, CA USA; 4 CoLabs, University of California, San Francisco, CA USA; 5 Northern California Institute for Research and Education (NCIRE), San Francisco, CA USA; 6 Bakar Computational Health Sciences Institute, University of California, San Francisco, CA USA; 7 Department of Pediatrics, University of California, San Francisco, CA USA

**Keywords:** Biomarkers, Computational biology and bioinformatics, Diseases, Neurology, Neuroscience

## Abstract

Leveraging multi-omics to better understand the molecular signatures and pathways underlying Alzheimer’s disease (AD) pathogenesis is critical for early diagnosis and disease modifying interventions. We performed peripheral blood transcriptome (*N* = 669) and epigenome microarray analyses (*N* = 553) on non-Hispanic white participants from the Alzheimer’s Disease Neuroimaging Initiative (ADNI) to identify molecular signatures of AD. We identified specific transcripts (e.g., *MAPK14*, *GM2A*, *CD177*) and co-expression networks that were dysregulated in AD, marked by a strong influence of *APOE* ε4 genotype, and characterized by a consistent pattern of immune activation, inflammation, and metabolic suppression. Further, these peripheral signatures were linked to central AD pathology (amyloid PET, CSF p-tau181) and neurodegeneration (plasma NfL, regional atrophy), with two genes, *MXD3* and *NR4A1*, identified as protective against progression from MCI to AD. Our work emphasizes the importance of *APOE* genotypes in AD pathophysiology and highlights potential targets for biomarker discovery and personalized therapeutic strategies.

## Introduction

Alzheimer’s disease (AD) is a chronic neurodegenerative disorder and the leading cause of dementia globally^1^. Pathologically, it is characterized by the progressive deposition of amyloid-beta (Aβ) plaques and neurofibrillary tau aggregates, followed by neurodegeneration and eventual cognitive impairment^2^. AD pathology begins to accumulate several years prior to clinical symptom onset, and the duration of exposure to amyloid accumulation is associated with accelerated cognitive decline and increased risk of AD^3^. Importantly, the extent of age and AD-related changes, particularly onset, progression trajectories, clinical manifestations, and biomarker phenotypes, varies from person to person. While neuroimaging has advanced our ability to detect structural atrophy (cortical and subcortical gray matter tissue loss), synaptic dysfunction, and pathological protein aggregation (Aβ and tau)^4^, significant gaps remain in understanding the molecular mechanisms that drive the heterogeneity of disease and pathology onset, progression trajectories, and patient-specific phenotypic manifestations^5,6^. Elucidating the biological pathways underlying this heterogeneity is critical for developing precision medicine approaches.

Peripheral blood transcriptomics and epigenetics offer accessible, multi-system insights into the molecular vulnerabilities driving AD. Emerging evidence suggests that peripheral changes reflect central neuropathology through mechanisms such as immune activation, blood-brain barrier (BBB) breakdown, and exosomal transport of extracellular vesicles^7–9^. While studies have identified alterations in DNA methylation (DNAm) and messenger ribonucleic acid (mRNA) gene expression in AD^10–13^ separately, integrating these two “omics” layers provides a more comprehensive view of how epigenetic modifications may regulate transcriptional output to drive disease phenotypes. However, it remains unclear how these peripheral molecular signatures relate to the longitudinal trajectories of specific AD pathologies, such as amyloid and tau accumulation and neurodegeneration.

Furthermore, AD pathogenesis is heavily influenced by genetic and biological risk factors, most notably *APOE* ε4 status and sex^14^. Sexual dimorphism in AD prevalence and progression is well-documented^15,16^, with women exhibiting higher prevalence and faster cognitive decline than men, yet the sex-specific molecular drivers in the periphery remain under-explored. Similarly, the *APOE* ε4 allele is the strongest genetic risk factor for sporadic AD^17,18^, but the extent to which peripheral immune and inflammatory signatures are independent of, or driven by, *APOE* genotype requires clarification.

**Fig. 1:**
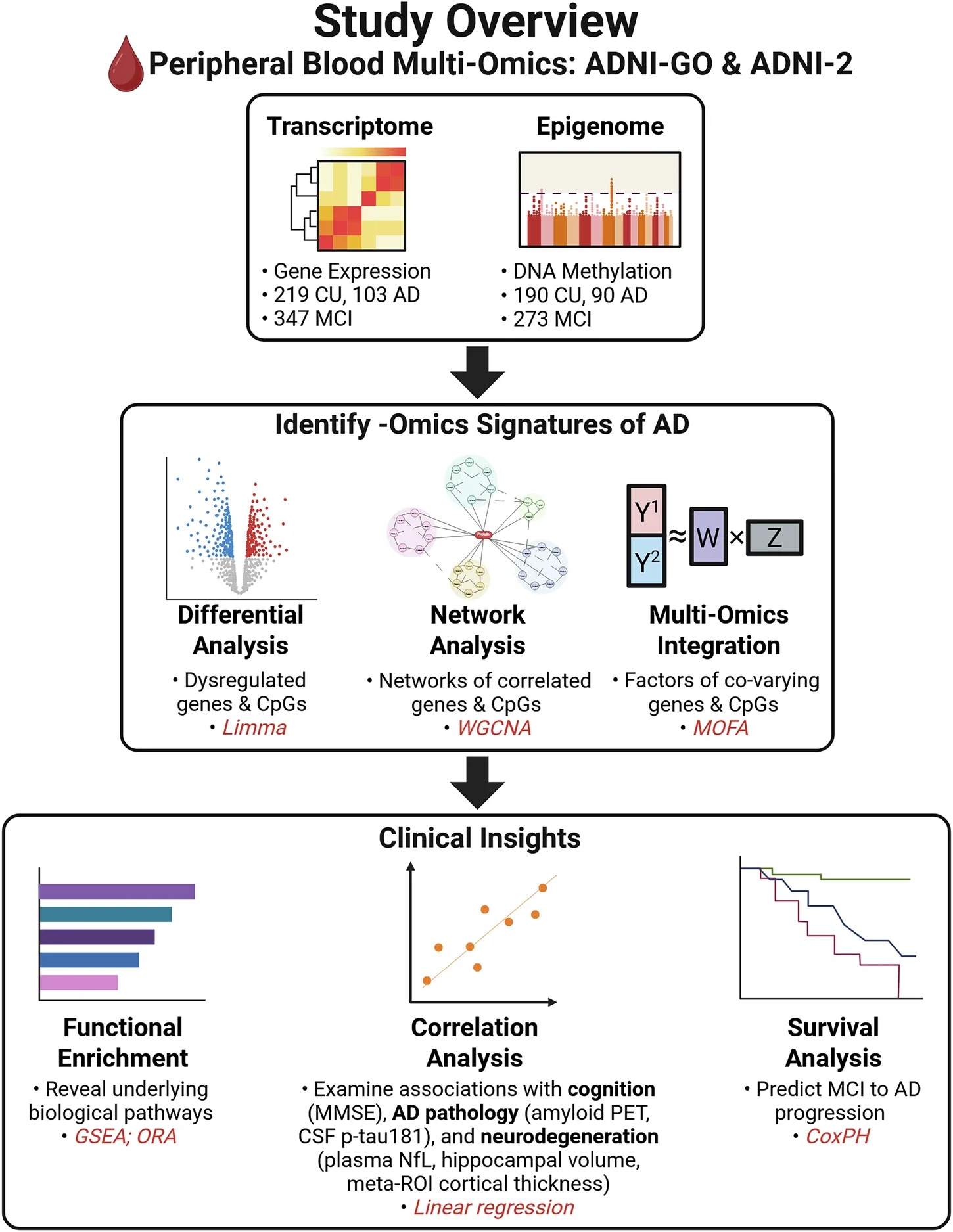
Study overview. Flowchart depicting the study overview including all datasets and analyses performed. Blood multi-omics data from non-Hispanic white participants were obtained from ADNI-GO/2 cohorts. CU cognitively unimpaired, MCI mild cognitive impairment, AD Alzheimer’s disease, WGCNA weighted gene co-expression network analysis, MOFA multi-omics factor analysis, GSEA gene set enrichment analysis, ORA overrepresentation analysis, MMSE mini-mental state examination, PET positron emission tomography, CSF cerebrospinal fluid, p-tau181 phosphorylated tau181, NfL neurofilament light chain, meta-ROI meta-region of interest, CoxPH Cox proportional hazards.

In this study, we leveraged multi-omics data from the Alzheimer’s Disease Neuroimaging Initiative (ADNI) to identify peripheral molecular signatures associated with AD. To minimize confounding due to population stratification, we focused our analysis on non-Hispanic white (NHW) participants. We examined whole-genome peripheral blood gene expression and DNAm profiles using microarrays to identify dysregulated genes, co-expression networks, and multi-omics factors (Fig. 1). We hypothesized that peripheral immune dysregulation is not merely a bystander but correlates longitudinally with central AD pathology accumulation (i.e., amyloid and tau) and neurodegeneration markers (e.g., neurofilament light chain [NfL], atrophy). By characterizing these signatures in the context of sex and *APOE* ε4 genotype, this study aims to facilitate the development of accessible biomarkers for early diagnosis and disease-modifying interventions.

## Results

### Participant characteristics

We analyzed a subset of self-reported NHW participants from the ADNI cohort to minimize population stratification confounders. The final analytic samples included 669 participants (219 cognitively unimpaired [CU], 347 with mild cognitive impairment [MCI], and 103 AD) with baseline gene expression data and 553 participants (190 CU, 273 MCI, and 90 AD) with baseline DNAm data. The demographic and clinical characteristics are detailed in Table 1a, b.

**Table 1:** Participant demographics and clinical characteristics

a. Non-Hispanic white participants with available gene expression data					
		Clinical Diagnosis			
Characteristic	Overall *N* = 669	CU *N* = 219	MCI *N* = 347	AD *N* = 103	*p*-value
Age, years	75 (8)	77 (6)	73 (8)	77 (8)	<0.001
Women, *n* (%)	291 (43%)	109 (50%)	145 (42%)	37 (36%)	0.042
*APOE* ε4 carrier, *n* (%)	264 (39%)	57 (26%)	140 (40%)	67 (65%)	<0.001
Education attainment, *n* (%)	-	-	-	-	0.123
High school or less	102 (15%)	22 (10%)	61 (18%)	19 (18%)	-
College	275 (41%)	98 (45%)	135 (39%)	42 (41%)	-
Postgraduate	292 (44%)	99 (45%)	151 (44%)	42 (41%)	-
RIN	6.89 (0.63)	6.85 (0.57)	6.92 (0.63)	6.88 (0.72)	0.427
Amyloid PET-positive, *n* (%)	278 (47%)	55 (29%)	160 (50%)	63 (81%)	<0.001
MCI-to-AD progressor, *n* (%)	88 (27%)	-	88 (27%)	-	-
CDR-SB	1.67 (2.31)	0.07 (0.29)	1.45 (1.02)	5.84 (2.82)	<0.001
MMSE	27.33 (3.47)	29.08 (1.24)	28.03 (1.69)	21.18 (4.47)	<0.001
Amyloid PET, centiloid	35 (47)	16 (36)	38 (48)	68 (49)	<0.001
CSF p-tau181, pg/mL	26 (13)	23 (9)	26 (13)	36 (15)	<0.001
Plasma NfL, pg/mL	43 (25)	42 (27)	41 (22)	55 (24)	<0.001
Hippocampal volume, mm^3^	7,039 (1,054)	7,498 (758)	7,020 (1,032)	5,678 (823)	<0.001
Meta-ROI cortical thickness, mm	2.74 (0.18)	2.79 (0.13)	2.74 (0.18)	2.48 (0.17)	<0.001

b. Non-Hispanic white participants with available DNA methylation data within ±1 year of gene expression profiling					
		Clinical diagnosis			
Characteristic	Overall *N* = 553	CU *N* = 190	MCI *N* = 273	AD *N* = 90	*p*-value
Age, years	75 (8)	77 (6)	73 (8)	77 (8)	<0.001
Women, *n* (%)	245 (44%)	93 (49%)	118 (43%)	34 (38%)	0.188
*APOE* ε4 carrier, *n* (%)	223 (40%)	46 (24%)	116 (42%)	61 (68%)	<0.001
Education attainment, *n* (%)	-	-	-	-	0.278
High school or less	77 (14%)	19 (10%)	42 (15%)	16 (18%)	-
College	232 (42%)	87 (46%)	107 (39%)	38 (42%)	-
Postgraduate	244 (44%)	84 (44%)	124 (45%)	36 (40%)	-
Amyloid PET-positive, *n* (%)	231 (47%)	46 (27%)	129 (50%)	56 (86%)	<0.001
MCI-to-AD progressor, *n* (%)	79 (30%)	-	79 (30%)	-	-
CDR-SB	1.70 (2.33)	0.07 (0.26)	1.44 (0.98)	5.88 (2.62)	<0.001
MMSE	27.24 (3.56)	29.05 (1.24)	28.03 (1.67)	21.03 (4.39)	<0.001
Amyloid PET, centiloid	34 (46)	15 (36)	37 (46)	73 (45)	<0.001
CSF p-tau181, pg/mL	26 (13)	23 (9)	26 (13)	37 (15)	<0.001
Plasma NfL, pg/mL	43 (24)	42 (27)	40 (19)	55 (25)	<0.001
Hippocampal volume, mm^3^	7054 (1,062)	7497 (723)	7052 (1,046)	5594 (777)	<0.001
Meta-ROI cortical thickness, mm	2.74 (0.18)	2.79 (0.12)	2.74 (0.18)	2.49 (0.17)	<0.001

Summary statistics of mean (SD) or *n* (%) based on complete observations are shown for study participants by clinical diagnosis with *p*-values from ANOVA (continuous traits) or chi-square (categorical values) to assess differences between the diagnostic groups.

*CU* cognitively unimpaired, *MCI* mild cognitive impairment, *AD* Alzheimer’s disease, *APOE* ε4 apolipoprotein E4, *RIN* RNA integrity number, *PET* positron emission tomography, *CDR-SB* clinical dementia rating sum of boxes, *MMSE* mini-mental state exam, *CSF* cerebrospinal fluid, *p-tau181* phosphorylated tau 181, *NfL* neurofilament light chain, *meta-ROI* meta-region of interest.

Consistent with established disease patterns, participants with AD were significantly more likely to be *APOE* ε4 carriers, amyloid PET positive, and exhibited greater cognitive impairment and neurodegeneration (lower hippocampal volume and elevated plasma NfL) compared to CU and MCI groups (Table 1a, b). Cohort characteristics, including counts of missing data for key variables and rates of change for all AD endophenotypes, are provided in Supplementary Table S1a, b. Missingness was handled using a complete-case approach in which observations with missing data were excluded from the corresponding analyses.

### Blood transcriptome microarray analysis reveals immune dysregulation, partially driven by *APOE*

**Fig. 2:**
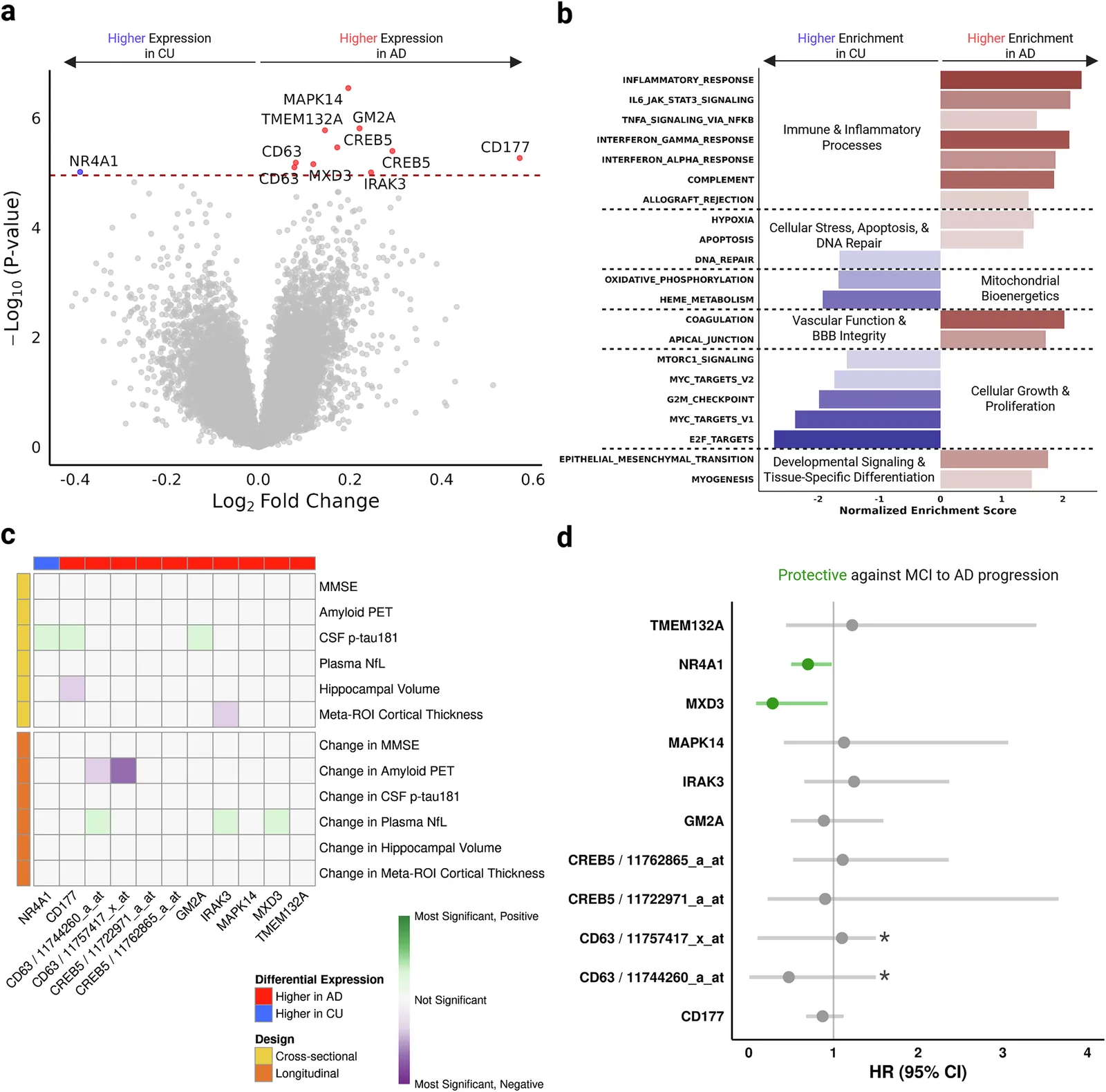
Microarray-based transcriptomic signatures of AD. **a** Volcano plot representing the top differentially expressed genes in AD that met the significance threshold of BH-FDR < 0.05 (dashed line). Red dots represent the upregulated genes, blue dots represent downregulated genes, and gray dots indicate that the genes did not meet the significance threshold. **b** Bar plot showing the top enriched Hallmark pathways (BH-FDR < 0.05). Red bars represent pathways with increased enrichment in AD relative to CU, while blue bars represent pathways with decreased enrichment in AD relative to CU. The shade of the bar denotes the degree of significance (i.e., the darker the shade the more significant; BH-FDR = 0–0.001, BH-FDR = 0.001–0.01, BH-FDR = 0.01–0.05). **c** Heatmap of associations between AD-related genes and AD endophenotypes, separated by cross-sectional and longitudinal design. Green indicates a significant positive association, while purple indicates a significant negative association. The shading denotes the degree of significance (*p* = 0–0.001, *p* = 0.001–0.01, *p* = 0.01–0.05). **d** Hazard ratio plot of the AD-related genes for predicting MCI to AD progression. Green indicates a significant protective effect against MCI to AD progression (*p* < 0.05), while gray indicates that the gene did not meet the significance threshold. Asterisk signifies that an artificial HR estimate and upper limit of the 95% CI were used for visualization only.

We first identified differentially expressed genes (DEGs) between AD and CU participants. Using a linear model adjusted for age, sex, education, RNA integrity number (RIN), and batch, we identified 11 transcripts mapping to nine unique genes significantly dysregulated in AD: 10 transcripts (mitogen-activated protein kinase 14 [*MAPK14*], ganglioside GM2 activator [*GM2A*], transmembrane protein 132 A [*TMEM132A*], cAMP responsive element binding protein 5 [*CREB5*], *CD63*, *CD177*, MAX dimerization protein 3 [*MXD3*], and interleukin 1 receptor associated kinase 3 [*IRAK3*]) were upregulated and one transcript, nuclear receptor subfamily 4 group A member 1 (*NR4A1*), was downregulated in AD (Benjamini-Hochberg false discovery rate (BH-FDR) < 0.05; Fig. 2a).

Gene set enrichment analysis (GSEA) revealed distinct biological signatures. Genes upregulated in AD were broadly enriched in pathways related to immune and inflammatory processes (e.g., inflammatory response, IL-6/JAK/STAT3 signaling, TNF-α signaling via NF-*κ*B, type I and II interferon responses, the complement cascade, and allograft rejection), cellular stress (e.g., hypoxia), apoptosis, vascular function and BBB integrity (e.g., coagulation and apical junction), developmental signaling (e.g., epithelial mesenchymal transition), and tissue-specific differentiation (e.g., myogenesis). Conversely, genes downregulated in AD were enriched in pathways related to DNA repair, mitochondrial bioenergetics (e.g., oxidative phosphorylation and heme metabolism), and cellular growth and proliferation (e.g., E2F targets, MYC targets, and G2/M checkpoint) (BH-FDR < 0.05; Fig. 2b, Supplementary Fig. S1).

Given that *APOE* ε4 is a major risk factor for AD, we performed a sensitivity analysis adjusting for *APOE* ε4 carrier status. Notably, the number of significant DEGs dropped from nine unique genes to three. Only *MAPK14*, *GM2A*, and *CD177* remained significantly upregulated in AD (BH-FDR < 0.05; Table 2). This suggests that a substantial portion of the identified microarray-based transcriptomic signature is *APOE* genotype-dependent. However, even after adjustment, upregulated genes remained enriched in immune and inflammatory processes, cellular stress, apoptosis, lipid metabolism (e.g., cholesterol homeostasis), vascular function and BBB integrity, developmental signaling, and tissue-specific differentiation (BH-FDR < 0.05; Supplementary Fig. S2a).

**Table 2:** Table showing the differential expression of the 11 identified AD-related genes after accounting for *APOE* ε4 genotype

Transcript	Log2FC [95% CI]	*p*-value	BH-FDR
*GM2A*/11719922_a_at	0.246 [0.154, 0.339]	2.16 × 10^−7^	**0.011**
*CD177*/11730765_at	0.626 [0.374, 0.879]	1.37 × 10^−6^	**0.034**
*MAPK14*/11742204_a_at	0.187 [0.110, 0.264]	2.13 × 10^−6^	**0.035**
*CREB5*/11762865_a_at	0.297 [0.169, 0.424]	6.35 × 10^−6^	0.071
*MXD3*/11746877_a_at	0.123 [0.069, 0.176]	9.18 × 10^−6^	0.071
*CD63*/11757417_x_at	0.083 [0.047, 0.120]	9.52 × 10^−6^	0.071
*TMEM132A*/11723445_a_at	0.138 [0.077, 0.199]	1.15 × 10^−5^	0.071
*CD63*/11744260_a_at	0.080 [0.044, 0.115]	1.16 × 10^−5^	0.071
*NR4A1*/11717994_a_at	-0.390 [-0.567, -0.212]	1.85 × 10^−5^	0.096
*CREB5*/11722971_a_at	0.164 [0.089, 0.238]	1.96 × 10^−5^	0.096
*IRAK3*/11742017_a_at	0.244 [0.132, 0.356]	2.30 × 10^−5^	0.096

Bold indicates significance at BH-FDR < 0.05.

In sex-stratified analyses, no individual transcripts met the significance threshold after correcting for multiple comparisons except for *GM2A* in men after accounting for *APOE* ε4 carrier status. However, exploratory GSEA of nominally significant genes suggested distinct biological drivers (Supplementary Fig. S3): unique enrichment in vascular function and BBB integrity pathways in men, and unique enrichment in cellular stress pathways in women. Both sexes showed shared increased enrichment in immune and inflammatory processes, and decreased enrichment in pathways related to mitochondrial bioenergetics and cellular growth and proliferation.

In a sensitivity analysis restricted to a subset of participants with diagnoses biologically confirmed by amyloid PET, all 11 transcripts remained nominally significant and showed strong effect size concordance with the main analysis (*r* = 0.82; *p* < 0.001; Supplementary Fig. S4). These findings suggest that the identified microarray-based transcriptomic signature is robustly linked to AD pathophysiology.

### Microarray-based transcriptomic signatures are linked to AD endophenotypes and progression

We then investigated whether the 11 identified DEGs were associated with AD-related endophenotypes independent of clinical diagnosis (Fig. 2c). At baseline, higher expression of *GM2A*, *CD177*, and *NR4A1*, was associated with greater CSF phosphorylated tau (p-tau181) levels (*GM2A*: *β* [95% CI] = 4.804 [0.514, 9.094], *p* = 0.028; *CD177*: *β* = 1.958 [0.394, 3.521], *p* = 0.014; *NR4A1*: *β* = 2.415 [0.244, 4.586], *p* = 0.029). Higher *CD177* expression was associated with lower hippocampal volume (*β* = −142.652 [−270.409, −14.895], *p* = 0.029), and higher *IRAK3* expression was associated with thinner meta-region of interest (meta-ROI) cortex (*β* = −0.053 [−0.104, −0.002], *p* = 0.042).

Longitudinally, we observed divergent associations with pathology versus neurodegeneration markers (Fig. 2c). Specifically, higher expression of *CD63* was associated with a faster rate of amyloid accumulation (at probe 11757417_x_at: *β* = −2.923 [−4.883, −0.962], *p* = 0.004; at probe 11744260_a_at: *β* = −2.475 [−4.504, −0.446], *p* = 0.017). Conversely, higher expression of *MXD3*, *IRAK3*, and *CD63* was associated with a slower rate of plasma NfL increase (*MXD3*: *β* = 1.069 [0.132, 2.006], *p* = 0.025; *IRAK3*: *β* = 0.517 [0.076, 0.957], *p* = 0.022; *CD63* at probe 11744260_a_at: *β* = 1.517 [0.125, 2.909], *p* = 0.033).

In survival analyses adjusted for age, sex, education, clinical dementia rating sum of boxes (CDR-SB), RIN, and batch, higher baseline expression of *MXD3* and *NR4A1* was associated with a significantly lower risk of MCI-to-AD progression (*MXD3*: HR [95% CI] = 0.280 [0.084, 0.929], *p* = 0.038; *NR4A1*: HR = 0.699 [0.499, 0.977], *p* = 0.036; Fig. 2d, Supplementary Table S2).

After adjusting for *APOE* ε4 carrier status, associations between *GM2A*/*CD177* and CSF p-tau181/hippocampal volume remained significant (Supplementary Fig. S2b), but no genes met the significance threshold for predicting MCI-to-AD progression.

### Gene co-expression network analysis (WGCNA) identifies robust immune networks

**Fig. 3:**
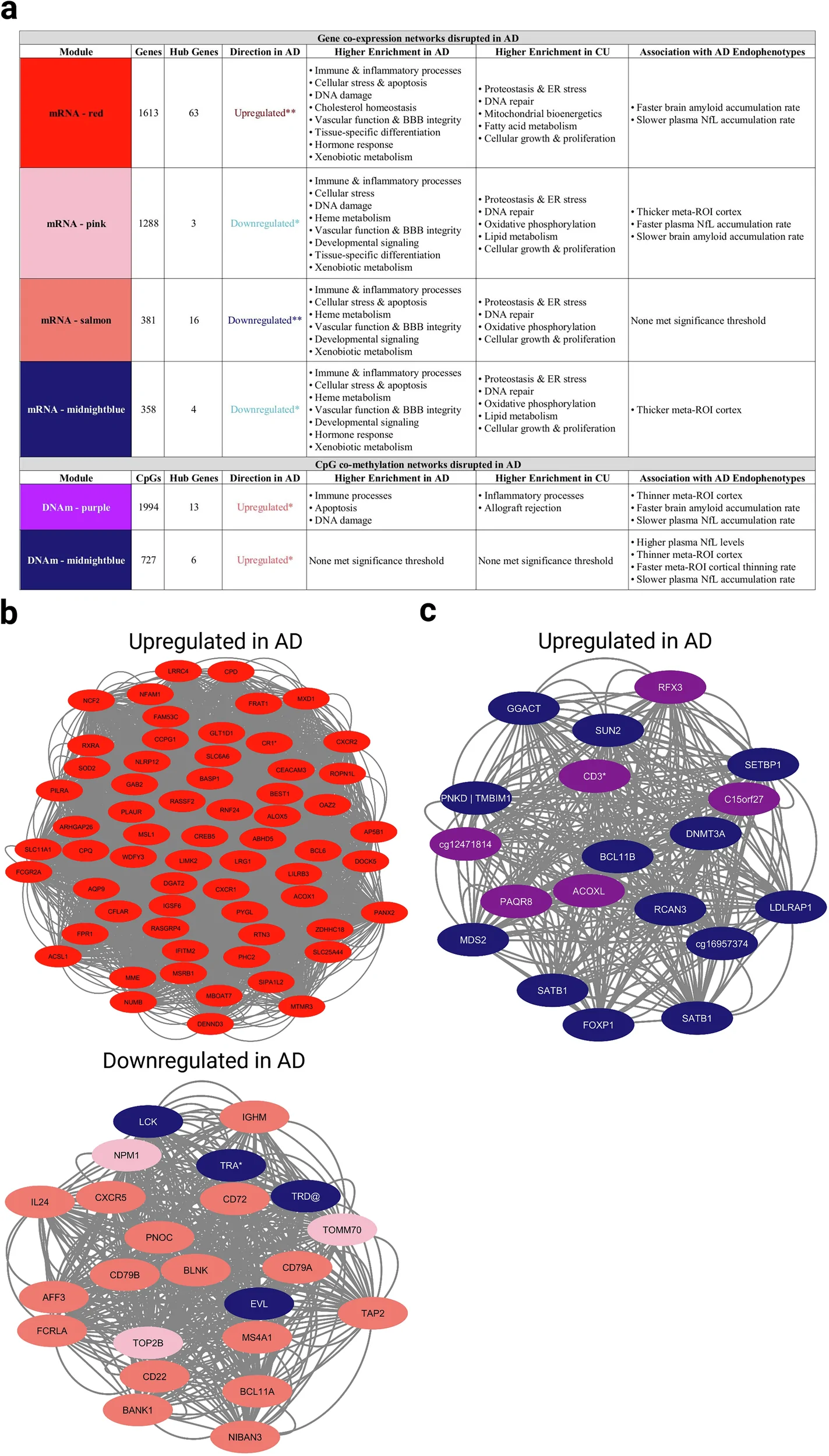
Dysregulated gene co-expression and CpG co-methylation networks in AD. **a** Table showing four gene co-expression networks and two CpG co-methylation networks that were significantly dysregulated in AD (*p* < 0.05). The number of genes, CpG sites, and hub genes within each network, categories for the top enriched pathways, and associations with AD endophenotypes are also shown. Asterisk and shading denote the degree of significance (***p = 0-0.001, **p = 0.001–0.01, *p = 0.01–0.05) and the color indicates whether the module was upregulated (red) or downregulated (blue) in AD. **b** Network plot of the hub genes in the up- and downregulated gene co-expression networks, grouped by direction in AD. CR1* is an abbreviation for *CR1* and *CR1L* which were both mapped to the same probe. TRA* is an abbreviation for *TRAC*, *TRAJ17*, and *TRAV20* which were mapped to the same probe. **c** Network plot of the hub genes in the two upregulated CpG co-methylation networks. CD3* is an abbreviation for *CD3D* and *CD3G* which were both mapped to the same probe.

To capture system-level changes, we performed weighted gene co-expression network analysis (WGCNA), after regressing out the effects of age, sex, education, RIN, and batch, identifying 37 distinct gene modules. Four modules (upregulated “mRNA - red” and downregulated “mRNA-pink,” “mRNA-salmon,” and “mRNA-midnightblue”) were significantly altered in AD (*p* < 0.05; Fig. 3a; Supplementary Fig. S5a). Using gene significance (GS) and module membership (MM) scores, we identified 63, 3, 16, and 4 hub genes in the “mRNA- red,” “mRNA-pink,” “mRNA-salmon,” and “mRNA-midnightblue” modules, respectively (GS < 0.05 and |MM| > 0.8; Fig. 3a, b).

GSEA revealed reciprocal enrichment patterns across AD-related modules. Genes in the “mRNA-red” module showed increased enrichment in AD for pathways related to immune and inflammatory processes, cellular stress, apoptosis, DNA damage (e.g., ultraviolet light response), cholesterol homeostasis, vascular function and BBB integrity, tissue-specific differentiation, hormone response (e.g., early estrogen response), and xenobiotic metabolism, while showing decreased enrichment in pathways related to proteostasis and ER stress, DNA repair, mitochondrial bioenergetics, fatty acid metabolism, and cellular growth and proliferation (BH-FDR < 0.05; Fig. 3a; Supplementary Fig. S6a). In contrast, genes in the downregulated modules (“mRNA-pink,” “mRNA-salmon,” and “mRNA-midnightblue”) showed increased enrichment in CU for pathways related to proteostasis and ER stress (e.g., unfolded protein response and protein secretion), DNA repair, mitochondrial bioenergetics, lipid metabolism, and cellular growth and proliferation, while showing decreased enrichment in pathways related to immune and inflammatory processes, cellular stress, apoptosis, vascular function and BBB integrity, developmental signaling, and xenobiotic metabolism (BH-FDR < 0.05; Fig. 3a; Supplementary Fig. S6b–d).

Unlike single-gene analysis, WGCNA revealed distinct sex-specific patterns. In men, one gene module, “mRNA-magenta,” was significantly upregulated in AD (*p* < 0.05; Supplementary Figs. S5b and S7a) and comprised of 12 hub genes (GS < 0.05 and |MM| > 0.8; Supplementary Fig. S7a, b), showing increased enrichment in AD for pathways related to immune and inflammatory processes, cellular stress, apoptosis, DNA damage, cholesterol homeostasis, vascular function and BBB integrity, developmental signaling, tissue-specific differentiation, and hormone response, and decreased enrichment in pathways related to proteostasis and ER stress, DNA repair, mitochondrial bioenergetics, fatty acid metabolism, and cellular growth and proliferation (BH-FDR < 0.05; Supplementary Figs. S7a and S8a). Women exhibited a more complex signature with five gene modules (upregulated “mRNA-darkturquoise,” “mRNA- darkolivegreen,” “mRNA-violet,” and “mRNA-plum1” and downregulated “mRNA-royalblue”) altered in AD (*p* < 0.05; Supplementary Figs. S5c and S7a). Hub gene analysis identified 15, 3, and 3 hub genes in the “mRNA-darkturquoise,” “mRNA-darkolivegreen,” and “mRNA-plum1” modules, respectively, while none met the criteria in the “mRNA-violet” and “mRNA- royalblue” modules (GS < 0.05 and |MM| > 0.8; Supplementary Fig. S7a–c). Genes in the upregulated modules showed increased enrichment in AD for immune and inflammatory processes, cellular stress, apoptosis, DNA damage, and vascular function and BBB integrity, while showing decreased enrichment in proteostasis and ER stress, DNA repair, and cellular growth and proliferation (BH-FDR < 0.05; Supplementary Figs. S7a and S8c–f). In contrast, the downregulated “mRNA-royalblue” module showed increased enrichment in CU for proteostasis and ER stress, DNA damage, cellular growth and proliferation, and hormone response, and decreased enrichment in immune and inflammatory processes, cellular stress, apoptosis, DNA repair, mitochondrial bioenergetics, vascular function and BBB integrity, and tissue-specific differentiation (BH-FDR < 0.05; Supplementary Figs. S7a and S8b).

To isolate effects independent of *APOE* genotype, we regressed out *APOE* ε4 carrier status prior to WGCNA. This analysis identified 38 distinct gene modules, 10 of which were significantly altered in AD (four upregulated and six downregulated modules; *p* < 0.05; Supplementary Figs. S9a and S10). Genes in both up- and downregulated modules showed broad enrichment in immune and inflammatory processes, proteostasis and ER stress, cellular stress, DNA damage, mitochondrial bioenergetics, lipid metabolism, cellular growth and proliferation, tissue-specific differentiation, and hormone response (BH-FDR < 0.05; Supplementary Figs. S10a and S11).

In sex-stratified and *APOE*-independent analysis, WGCNA identified 33 and 39 distinct gene modules in men and women, respectively. In men, two modules, “mRNA-grey60” and “mRNA- skyblue,” were significantly downregulated in AD (*p* < 0.05; Supplementary Figs. S9b and S12a), which were comprised of 19 and 3 hub genes, respectively (GS < 0.05 and |MM| > 0.8; Supplementary Fig. S12a, b). These modules showed nearly inverse enrichment in pathways involving immune and inflammatory processes, cellular stress, DNA damage, mitochondrial bioenergetics, lipid metabolism, vascular function and BBB integrity, developmental signaling, tissue-specific differentiation, and xenobiotic metabolism (BH-FDR < 0.05; Supplementary Figs. S12a and S13a, b). In women, four modules (upregulated “mRNA-darkgreen” and “mRNA-steelblue” and downregulated “mRNA-lightcyan” and “mRNA-royalblue”) were significantly altered in AD (*p* < 0.05; Supplementary Figs. S9c and S12a). Hub gene analysis identified 15, 1, 2, and 5 hub genes in the “mRNA-darkgreen,” “mRNA-steelblue,” “mRNA-lightcyan,” and “mRNA-royalblue” modules, respectively (GS < 0.05 and |MM | > 0.8; Supplementary Fig. S12a–c). Genes in the upregulated modules were enriched in AD for various pathways related to immune and inflammatory processes, proteostasis and ER stress, cellular stress, apoptosis, DNA damage, mitochondrial bioenergetics, lipid metabolism, vascular function and BBB integrity, and cellular growth and proliferation (BH-FDR < 0.05; Supplementary Figs. S12a and S13e, f). Uniquely, downregulated modules shared a signature of increased enrichment in CU for immune pathways and decreased enrichment in inflammation pathways. Additionally, they shared enrichment in pathways related to proteostasis and ER stress, cellular stress, DNA repair, mitochondrial bioenergetics, lipid metabolism, vascular function and BBB integrity, cellular growth and proliferation, tissue-specific differentiation, and hormone response (BH-FDR < 0.05; Supplementary Figs. S12a and S13c, d).

### AD-related gene modules are linked to AD endophenotypes

At baseline (Fig. 3a), thicker meta-ROI cortex was associated with higher expression of genes in the proliferation-driven “mRNA-pink” (*β* = 0.474 [0.073, 0.875], *p* = 0.021) and “mRNA-midnightblue” modules (*β* = 0.403 [0.009, 0.797], *p* = 0.045). Longitudinally (Fig. 3a), higher expression of genes in the immune response and inflammation-driven “mRNA - red” module was associated with a faster rate of brain amyloid accumulation (*β* = −6.578 [−11.766, −1.390], *p* = 0.013) and a slower rate of increase in plasma NfL levels (*β* = 5.138 [1.249, 9.026], *p* = 0.01). In contrast, higher expression of genes in the proliferation-driven “mRNA-pink” module was associated with a slower rate of brain amyloid accumulation (*β* = 6.01 [0.596, 11.424], *p* = 0.03) and a faster rate of increase in plasma NfL levels (*β* = −4.2 [−8.126, −0.274], *p* = 0.036).

When sex-stratified (Supplementary Fig. S7a), within men, the immune response and inflammation-driven “mRNA-magenta” module mirrored the general “mRNA-red” pattern in its association with a faster rate of brain amyloid accumulation (*β* = −6.812 [−11.883, −1.742], *p* = 0.009) and a slower rate of increase in plasma NfL levels (*β* = 5.551 [1.546, 9.557], *p* = 0.007). Within women, associations were primarily cognitive and structural. The immune response-driven “mRNA-plum1” module was associated with higher baseline mini-mental state examination (MMSE) (*β* = 6.603 [1.456, 11.751], *p* = 0.012). In contrast, the tissue-specific differentiation-driven “mRNA-darkturquoise” module tracked with impairment: lower baseline MMSE (*β* = −8.940 [−13.856, −4.023], *p* < 0.001) and a faster rate of cognitive decline (*β* = −0.949 [−1.857, −0.040], *p* = 0.041). The proliferation-driven “mRNA-royalblue” module was linked to higher baseline hippocampal volume (*β* = 2270.468 [9.345, 4531.591], *p* = 0.049), and the immune response and inflammation-driven “mRNA-violet” module was associated with a slower rate of increase in plasma NfL levels (*β* = 3.893 [0.102, 7.683], *p* = 0.044).

### *APOE*-independent associations of gene modules with AD endophenotypes

At baseline (Supplementary Fig. S10a), the tissue-specific differentiation-driven “mRNA- white” module was associated with lower MMSE (*β* = −8.06 [−13.401, −2.720], *p* = 0.003), while the immune response-driven “mRNA-skyblue3” module was linked to higher CSF p-tau181 (*β* = 30.934 [3.476, 58.392], *p* = 0.027) and thinner meta-ROI cortex (*β* = −0.487 [−0.870, −0.105], *p* = 0.013). Longitudinally, the immune response-driven “mRNA-skyblue3” was further associated with a faster rate of cortical thinning (*β* = −0.064 [−0.114, −0.013], *p* = 0.013). Consistent with the general “mRNA-red” pattern, the immune response and inflammation-driven “mRNA-yellow” module showed divergent pathology trends: a faster rate of brain amyloid accumulation (*β* = −6.961 [−12.105, −1.816], *p* = 0.008) but a slower rate of increase in plasma NfL levels (*β* = 5.332 [1.472, 9.191], *p* = 0.007).

Within men-only stratified analysis (Supplementary Fig. S12a), the proliferation-driven “mRNA-grey60” module appeared protective as it was associated with slower rate of accumulation of both brain amyloid (*β* = 5.431 [0.304, 10.557], *p* = 0.038) and CSF p-tau181 (*β* = 2.112 [0.628, 3.595], *p* = 0.006). Within women stratified analysis (Supplementary Fig. S12a), the tissue-specific differentiation-driven “mRNA-darkgreen” module showed mixed signals: it was associated with thicker baseline cortex (*β* = 1.140 [0.030, 2.249], *p* = 0.044) but lower baseline MMSE (*β* = −8.864 [−13.849, −3.880], *p* = 0.001) and a faster rate of cognitive decline (*β* = −1.063 [−1.980, −0.145], *p* = 0.023). Conversely, the immune response-driven “mRNA- steelblue” module was linked to higher baseline MMSE (*β* = 5.720 [0.561, 10.879], *p* = 0.03), and the proliferation-driven “mRNA-royalblue” module was associated with lower baseline amyloid burden (*β* = −86.158 [−169.024, −3.291], *p* = 0.042).

In survival analyses predicting progression from MCI to AD, none of the AD-related gene modules (primary, sex-specific, or *APOE*-independent) met the significance threshold.

### Blood epigenetic analysis reveals subtle DNA methylation changes in AD

We performed whole-genome differential methylation analysis in ADNI samples within one year of gene expression profiling (*N* = 553). Using a linear model adjusted for age, sex, education, cell-type composition, and batch, no individual CpG sites met the significance threshold after correcting for multiple comparisons. Similarly, no individual CpG sites in sex-stratified, *APOE*-independent, or amyloid PET-confirmed sensitivity analyses met the significance threshold after correcting for multiple comparisons.

### Co-methylation network analysis reveals immune-related dysregulation in AD

Given that individual CpG sites likely have overlapping epigenetic regulatory mechanisms and similar methylation patterns, we performed WGCNA to identify modules of co-methylated CpG sites dysregulated in AD. In brief, we constructed a co-methylation network from the top 50% most variable CpG sites (372,153 CpGs) after regressing out the effects of age, sex, education, cell-type composition, and batch, and identified 22 distinct CpG modules, two of which (“DNAm-purple” and “DNAm-midnightblue” modules) were significantly upregulated in AD (*p* < 0.05; Figs. 3a and S14a). Hub gene analysis identified 13 hub genes in the “DNAm- purple” module and 6 in the “DNAm-midnightblue” module (GS < 0.05 and |MM | > 0.8; Fig. 3a, c).

CpG sites in the upregulated “DNAm-purple” showed increased enrichment in AD for pathways related to immune processes, apoptosis, and DNA damage, while also showing decreased enrichment in immune and inflammatory processes (BH-FDR < 0.05; Fig. 3a; Supplementary Fig. S15a). None of the pathways in the “DNAm-midnightblue” module met the significance threshold.

In sex-stratified analyses, with or without *APOE*-adjustment, none of the methylation profiles of identified CpG modules met the significance threshold.

After regressing out the effect of *APOE* ε4 carrier status prior to WGCNA, we identified 22 distinct CpG modules, and of these, two modules, “DNAm-tan” and “DNAm-lightyellow,” were significantly upregulated in AD (*p* < 0.05; Supplementary Figs. S10a and S14b), which were comprised of 6 and 12 hub genes, respectively (GS < 0.05 and |MM| > 0.8; Supplementary Fig. S10a, c). Consistent with primary analyses, CpG sites in the upregulated “DNAm-tan” module showed increased enrichment in AD for pathways related to immune and inflammatory processes, apoptosis, and DNA damage, while also showing decreased enrichment in immune and inflammatory processes (BH-FDR < 0.05; Supplementary Figs. S10a and S15b). None of the pathways in the “DNAm-lightyellow” module met the significance threshold.

### AD-related CpG modules are linked to AD endophenotypes

At baseline (Fig. 3a), a thinner meta-ROI cortex was associated with higher methylation of CpG sites in the immune response and inflammation-driven “DNAm-purple” module (*β* = −0.408 [−0.802, −0.013], *p* = 0.043) and the “DNAm-midnightblue” module (*β* = −0.427 [−0.814, −0.040], *p* = 0.031). Higher methylation of CpG sites in the “DNAm-midnightblue” module was also associated with higher plasma NfL levels (*β* = 54.813 [1.945, 107.681], *p* = 0.042). Longitudinally (Fig. 3a), higher methylation of CpG sites in the immune response and inflammation-driven “DNAm-purple” module was associated with a faster rate of brain amyloid accumulation (*β* = −6.225 [−11.615, −0.835], *p* = 0.024) and a slower rate of increase in plasma NfL levels (*β* = 4.254 [0.331, 8.177], *p* = 0.034). Higher methylation of CpG sites in the “DNAm- midnightblue” module was associated with a faster rate of cortical thinning (*β* = −0.054 [−0.103, −0.005], *p* = 0.031) and a slower rate of increase in plasma NfL levels (*β* = 6.163 [2.235, 10.092], *p* = 0.002).

### *APOE*-independent associations of CpG modules with AD endophenotypes

Associations of *APOE*-independent CpG modules mirror the general CpG module patterns observed in the primary analyses. At baseline (Supplementary Fig. S10a), thinner meta-ROI cortex was associated with higher methylation of CpG sites in the immune response and inflammation-driven “DNAm-tan” module (*β* = −0.408 [−0.797, −0.019], *p* = 0.040) and the “DNAm-lightyellow” module (*β* = −0.449 [−0.835, −0.064], *p* = 0.023). Higher methylation of CpG sites in the “DNAm-lightyellow” module was also associated with higher plasma NfL levels (*β* = 58.974 [6.118, 111.830], *p* = 0.029). Longitudinally (Supplementary Fig. S10a), higher methylation of CpG sites in the immune response and inflammation-driven “DNAm-tan” module was associated with a faster rate of brain amyloid accumulation (*β* = −6.441 [−11.889, −0.993], *p* = 0.021) and a slower rate of increase in plasma NfL levels (*β* = 5.419 [1.507, 9.330], *p* = 0.007). Higher “DNAm-lightyellow” was associated with a faster rate of cortical thinning (*β* = −0.052 [−0.101, −0.004], *p* = 0.036) and a slower rate of increase in plasma NfL levels (*β* = 6.429 [2.514, 10.345], *p* = 0.001).

In survival analyses predicting progression from MCI to AD, none of the AD-related CpG modules (primary or *APOE*-independent) met the significance threshold.

### Multi-omics factor analysis reveals consistent dysregulated pathways in AD

**Fig. 4:**
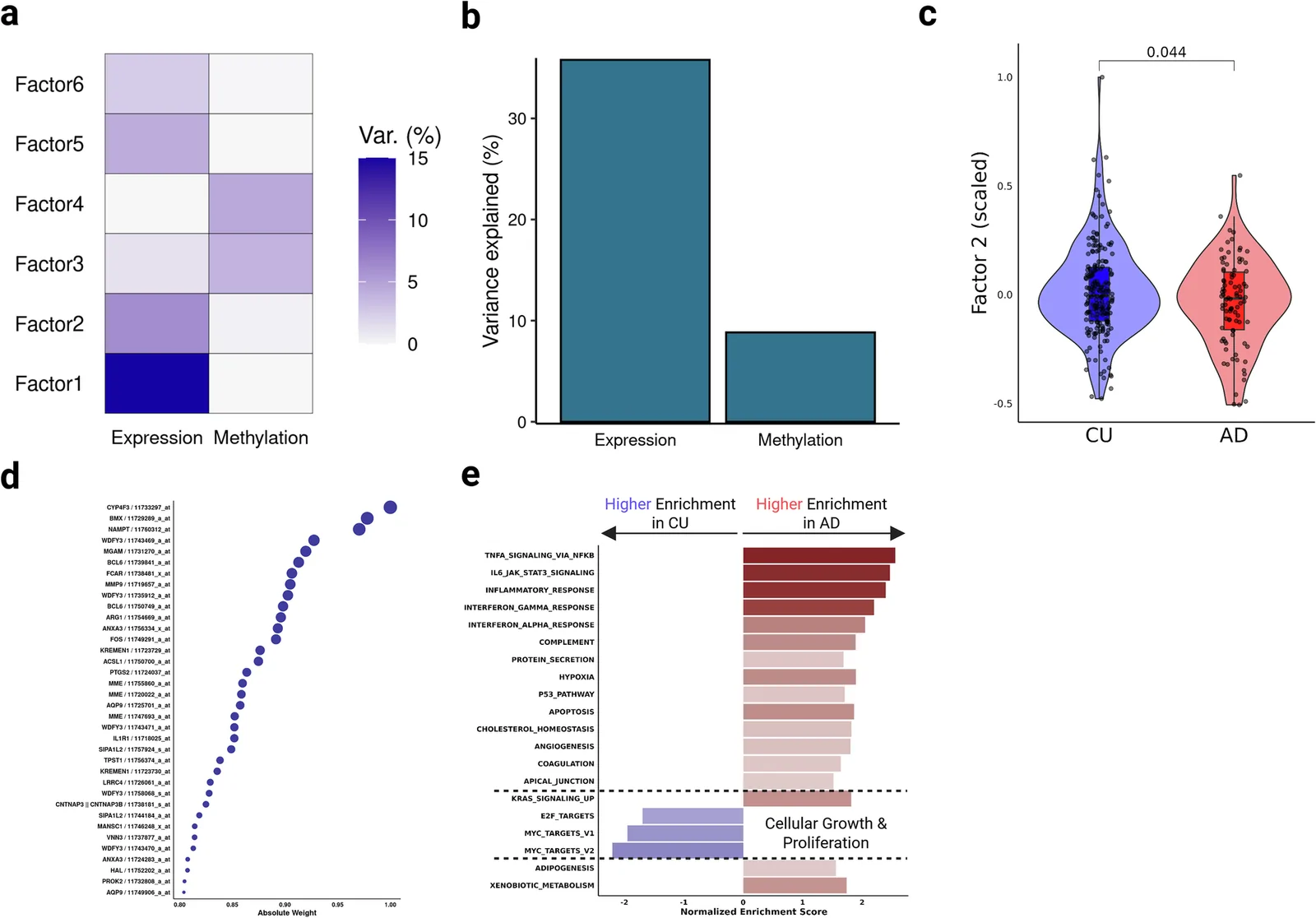
Multi-omics factor analysis identifies “Factor 2” as dysregulated in AD. **a** Variance explained plot in each -omics and learned latent factor. **b** Bar plot of the cumulative variance explained in each -omics across all factors. **c** Violin plot of “Factor 2” in CU individuals compared to AD individuals. A t-test was used to determine statistical significance between CU and AD samples. **d** Dot plot of the genes met the hub gene criteria of |weight| > 0.8. **e** Bar plot showing the top enriched Hallmark pathways (BH-FDR < 0.05) in “Factor 2”.

Finally, we performed multi-omics factor analysis (MOFA) to integrate transcriptomic and epigenetic microarray datasets (*n* = 280 paired samples) and to identify latent factors explaining shared biological variation. Prior to training the MOFA model, we regressed out the effects of sex, RIN, cell-type composition, and batch and selected the top 40% most variable genes (*n* = 19,717) and the top 3% most variable CpG sites (*n* = 22,329). After model training, MOFA identified six factors with a minimum explained variance of 2% (Fig. 4a). Gene expression was the primary driver of variance across factors (Fig. 4b). One of the axes of variation identified, “Factor 2,” was significantly downregulated in AD (*p* = 0.047; Fig. 4c); however, this association was attenuated after adjusting for age and education. The top hub genes (cytochrome P450 family 4 subfamily F member 3 [*CYP4F3*], bone marrow tyrosine kinase on chromosome X [*BMX*], nicotinamide phosphoribosyltransferase [*NAMPT*]; |weight| > 0.8; Fig. 4d) were enriched for cellular growth and proliferation (BH-FDR < 0.05; Fig. 4e), while contributing CpG sites showed decreased enrichment in an immune pathway (allograft rejection; *p* = 0.043). Higher expression of “Factor 2” was associated with a faster rate of increase in plasma NfL levels (*β* = −0.186 [−0.347, −0.025], *p* = 0.024), as well as a significantly lower risk of MCI-to-AD progression after adjusting for age, sex, education, and CDR-SB (HR = 0.006 [0.000, 0.838], *p* = 0.042).

In sensitivity analyses (sex-stratified or *APOE*-independent), none of the factors were significantly associated with AD diagnosis.

## Discussion

This study leveraged a well-characterized NHW subset of the ADNI cohort to identify peripheral molecular signatures associated with AD and AD-related endophenotypes. Our multi-omics analysis revealed a consistent upregulation of immune and inflammatory pathways and a downregulation of mitochondrial and metabolic pathways in the blood of patients with AD. While we identified specific transcripts (e.g., *MAPK14*, *GM2A*, *CD177*) and co-expression networks linked to central AD pathology (e.g., amyloid PET and CSF p-tau181) and neurodegeneration (e.g., plasma NfL and atrophy), a critical finding of this study is the strong influence of *APOE* ε4 genotype on these peripheral signatures. Furthermore, we identified that higher expression of two genes, *NR4A1* and *MXD3*, served as potential protective markers against clinical progression from MCI to AD. While *NR4A1* has been previously implicated in AD-related pathways^19,20^, the role of *MXD3* in AD remains poorly characterized and warrants further investigation^21^.

A major observation in our study was that the majority of the AD-associated, microarray-based transcriptomic signature appears to be driven by *APOE* genotype rather than disease status alone. Upon adjusting for *APOE* ε4 carrier status, the number of differentially expressed genes dropped from eleven to three (*MAPK14*, *GM2A*, *CD177*). This suggests that much of the peripheral immune dysregulation reported in AD literature^22–24^ may be a phenotypic readout of the *APOE* ε4 allele itself, which is known to heighten pro-inflammatory responses^25^, rather than a downstream consequence of amyloid or tau pathology. However, *MAPK14*, *GM2A*, and *CD177* remained significant even after adjustment, representing a robust, *APOE*-independent AD signature (Table 2). *MAPK14* is a crucial mediator of inflammatory signaling and immune-modulatory responses^26^. Knockout of *MAPK14* in mouse and cell models has been shown to attenuate amyloid plaque formation, decrease tau phosphorylation, downregulate pro-inflammatory cytokines, modulate neurogenesis, and improve memory^27–29^, demonstrating the potential of *MAPK14* inhibition as a disease-modifying therapy for AD. *GM2A* is of particular interest given its role in lysosomal function and lipid metabolism^30^; its elevation in blood mirrors findings in CSF and brain tissue, where it has been linked to amyloid plaque-associated neurotoxicity^31,32^. Similarly, *CD177*, a neutrophil activation marker, was associated with higher p-tau181 and hippocampal atrophy in our cohort. This aligns with evidence that BBB compromise in AD facilitates the infiltration of peripheral neutrophils, potentially exacerbating central neurodegeneration^33^.

Support for the immune exhaustion hypothesis^34^, in which microglia and infiltrating T-cells become dysfunctional (exhausted) from persistent pathology and paradoxically promote neuroinflammation, comes from our findings that *NR4A1* was significantly downregulated in AD and that the “DNAm-purple” co-methylation network was significantly upregulated in AD. *NR4A1* is an immediate-early gene critical for regulating T-cell exhaustion, inflammation control, and mitochondrial bioenergetics^35–38^. While *NR4A1* is often upregulated in postmortem brain tissue^19,20,39^, its downregulation in peripheral blood^40,41^ suggests a distinct systemic compartment effect—potentially a depletion or suppression of peripheral immune resilience. Notably, in our study, we found that higher baseline *NR4A1* was associated with elevated CSF p-tau181 levels and was protective against progression from MCI to AD. These findings suggest that maintaining *NR4A1* expression in the periphery, thereby promoting functional immune regulation and reducing inflammation, may confer resilience against clinical decline even in the presence of AD pathology. While single-site DNAm changes were subtle, co-methylation network analysis confirmed the microarray-based transcriptomic findings, identifying the immune response and inflammation-driven “DNAm - purple” module that was linked to longitudinal amyloid and NfL changes. The top hub CpG site within this module was located in the promoter region (i.e., within 1500 base pairs upstream of the transcription start site) of two genes, *CD3G* and *CD3D*, which encode essential subunits of the T-cell receptor complex required for T-cell signaling and activation^42^. The AD-associated hypermethylation of this promoter region suggests epigenetic suppression of *CD3G* and *CD3D*, potentially compromising T-cell surface expression and activation, which may contribute to T-cell exhaustion, impaired amyloid clearance, and neuroinflammation.

In addition to immune and inflammation dysregulation, our analysis highlighted a robust downregulation of proteostasis and ER stress, mitochondrial bioenergetics, and cellular growth and proliferation pathways in AD (e.g., “mRNA-pink,” “mRNA-salmon,” and “mRNA-midnightblue” modules). This aligns with the “bioenergetic failure” or “Type 3 Diabetes” hypothesis of AD, where mitochondrial dysfunction and glucose hypometabolism precipitate neuronal death^43–46^. Indeed, the top hub gene in the proliferation-driven “mRNA-pink” module was *NPM1* (nucleophosmin 1), which plays a vital role in preventing protein aggregation and mediating protein folding^47^, regulating mitochondrial energy metabolism and health^48^, repairing DNA damage^49^, and promoting cell cycle progression^50^, and has been found to be downregulated in postmortem AD brain tissue^51^. Additionally, a top hub gene in the multi-omics “Factor 2” was *NAMPT*, a key regulator of NAD^+^ biosynthesis and mitochondrial health^52,53^. The contribution of these hub genes to the downregulated signatures of AD supports the view that systemic metabolic failure is a core feature of AD pathogenesis, parallel to immune and inflammation dysregulation.

Although single-gene expression analyses showed no significant sex differences, co-expression network analyses revealed that women exhibited a distinct and more complex molecular signature of AD than men (Supplementary Figs. S7 and S12). Specifically, we identified multiple dysregulated modules linked to cognitive resilience (e.g., immune response-driven “mRNA-plum1” and “mRNA-steelblue” modules associated with higher MMSE), neuroprotection (e.g., the proliferation-driven “mRNA-royalblue” module associated with higher hippocampal volume and immune response and inflammation-driven “mRNA-violet” module associated with slower plasma NfL accumulation rate), and cognitive vulnerability (e.g., tissue-specific differentiation-driven “mRNA-darkturquoise” and “mRNA-darkgreen” modules associated with lower MMSE and faster cognitive decline). These findings suggest that the molecular drivers of AD, or the compensatory mechanisms combating it, may differ fundamentally by sex, warranting sex-stratified approaches in future biomarker development.

Our network analyses revealed instances of modular heterogeneity, where distinct gene co-expression modules moving in the same overall direction in AD exhibited opposing enrichment for the same broad biological pathways (e.g., TNF-α signaling via NF-*κ*B). This suggests that peripheral immune dysregulated in AD is not monolithic; rather, one module may capture a compensatory, hyperactive immune state, while another reflects isolated pathways undergoing immune exhaustion or suppression. Notably, these opposing enrichment patterns were most pronounced in our *APOE*-independent networks. Since *APOE* ε4 is known to drive a dominant, uniform pro-inflammatory signal, regressing out its effects likely unmasks the highly heterogeneous, underlying immune architecture of AD. This aligns with recent transcriptomic evidence in the blood and brain demonstrating that the activation of specific immune and inflammatory pathways in AD is highly dependent on *APOE* genotype^54^.

This study has several limitations. First, the cohort was restricted to non-Hispanic White participants to minimize population stratification, limiting generalizability. Second, the differential methylation signal was weak and inconclusive, likely due to the strict multiple testing correction in a moderate sample size. Third, while we examined longitudinal endophenotypes, the -omics data itself was cross-sectional; future studies should track longitudinal changes in gene expression to separate state-markers from trait-markers. Finally, the MOFA analysis was driven primarily by gene expression variance, highlighting the technical challenges of integrating multi-omics data with vastly different dimensionalities.

In summary, our multi-omics analysis identifies a peripheral signature of AD characterized by immune activation and metabolic suppression. We demonstrate that a significant portion of the peripheral AD profile is driven by *APOE* ε4 status, underscoring the need to account for genotype in blood-based biomarker studies. The identification of *APOE*-independent markers like *GM2A* and *CD177*, alongside protective factors like *NR4A1*, offers new targets for understanding the systemic biology of AD and developing precision medicine interventions.

## Methods

### Study design and participants

Data were obtained from the publicly available Alzheimer’s Disease Neuroimaging Initiative (ADNI) database (https://adni.loni.ucsf.edu), which was launched in 2003. The primary goal of the ADNI is to examine whether serial magnetic resonance imaging (MRI), PET, other biological markers, and clinical and neuropsychological assessment can be combined to measure the progression of MCI and early AD. The participant pool consists of CU individuals, individuals with MCI, and individuals clinically diagnosed with dementia due to AD. Inclusion and exclusion criteria for ADNI have been described in detail elsewhere^55^. All ADNI research activities were approved by the Advarra Institutional Review Boards for the US (IRB reference: Pro00064250) and all participants provided written consent.

This study leveraged data from ADNI-GO and ADNI-2 cohorts with available peripheral blood gene expression and DNAm profiling by microarrays, cognitive assessments, amyloid PET and MRI, and plasma and CSF biomarkers data. For the present study, the time of gene expression profiling was considered “baseline.” We utilized the closest DNAm, clinical, and biomarker assessments within ±1 year of the baseline gene expression profiling. Our analytic sample focused on a subset of self-reported NHW participants to reduce the risk of confounding due to race and ethnicity. A total of 669 participants (219 CU, 347 MCI, and 103 AD) and 553 participants (190 CU, 273 MCI, and 90 AD) had gene expression and DNAm profiling, respectively (Table 1a, b). *APOE* genotyping was performed as part of the standard ADNI protocol.

### Gene expression profiling

Gene expression profiling from peripheral blood samples collected using PAXgene tubes was performed on the Affymetrix Human Genome U219 Array (www.affymetrix.com, Santa Clara, CA), containing 530,467 probes for 49,293 transcripts. All probe sets were mapped and annotated with reference to the human genome (hg19). Quality control (QC) procedures on samples and probe sets were performed by the ADNI Genetics Core, as described elsewhere^13^. In brief, raw microarray expression values underwent Robust Multi-chip Average (RMA) normalization, sample sex and identity verification^56,57^, and three questionable samples were removed. Following the exclusion of 93 Affymetrix internal control probes, the final gene expression profiles contained 49,293 probes in 669 NHW participants with no missingness (Supplementary Fig. S16a).

In gene expression analyses, it is necessary to account for the RNA quality of each sample, as measured by RIN. Values of RIN range from 1-10, with higher values indicating greater RNA integrity and lower values indicating greater RNA degradation (i.e., lower RNA integrity). RIN has been found to be significantly lower in dementia patients relative to healthy controls, reinforcing the importance of controlling for RNA quality in the context of AD^58^. Therefore, RIN was used as a covariate in our analyses.

### DNA methylation profiling

Whole-genome DNAm profiling from peripheral blood samples was performed at AbbVie, Inc. on the Infinium MethylationEPIC BeadChip microarray (Illumina, Inc., San Diego, CA, USA), which covers ~866,000 CpG sites. Genomic DNA samples obtained from the National Centralized Repository for Alzheimer’s Disease and Related Dementias (NCRAD) were bisulfite-converted using the EZ-DNA Methylation kits (Zymo Research, Irvine, CA, USA) and subsequently analyzed using the Illumina Infinium HD methylation protocol on the HiScan system (Illumina, Inc.). QC procedures on samples were performed by AbbVie and the ADNI Genetics Core independently, as described in detail previously^10,11^, resulting in 1905 longitudinal samples for 649 participants.

Raw DNAm from all 1905 samples was normalized using the dasen method in the *wateRmelon* R package^59^ and converted to beta-values. We performed probe-level QC that excluded CpG probes with a detection *p* > 0.05 across all samples via the *minfi* R package^60^, nearby to single nucleotide polymorphisms (within 2 base-pairs and with a minor allele frequency >0.05), cross-hybridizing probes, and probes located on sex chromosomes via the rmSNPandCH function in the *DMRcate* R package^61^. We further excluded non-CpG methylation sites and CpGs without genomic annotations. Following the removal of technical replicates and samples with bisulfite conversion rates below 85%, the final DNAm profiles contained 744,306 probes in 553 NHW participants with no missingness (Supplementary Fig. S16b).

Since DNA methylation varies greatly between cell types in peripheral blood^62^, it is necessary to account for cell-type proportions as covariates in our models. Reference-based deconvolution was used to estimate immune cell-type composition (neutrophils, monocytes, eosinophils, NK cells, B cells, CD4+ T cells, and CD8+ T cells) from the bulk methylation data via the *EpiDISH* R package^63^. Six of seven estimated cell types were used as covariates, with eosinophils excluded to avoid multicollinearity.

### Cognitive performance

Participants undergo a comprehensive neuropsychological battery at clinical visits to examine global cognition, memory, executive function, attention, visuospatial ability, and language^55^. In this study, two cognitive measures were considered: MMSE was used as a measure of global cognitive function, and CDR-SB was used as a measure of cognitive and functional impairment severity.

### Amyloid PET and MRI-based atrophy measures

Amyloid PET imaging used ^18^F-florbetapir (FBP) with standardized ADNI protocols^64^. Global cortical standardized uptake value ratio (SUVR) was calculated by averaging PET uptake across cortical regions (frontal, cingulate, parietal, and lateral temporal). Amyloid PET SUVRs were converted to Centiloids (CL), a standardized measure of total amyloid burden ranging from 0 to 100, by the ADNI PET Core using tracer-specific equations as described previously^65,66^. A previously established and validated amyloid positivity threshold was set at the upper 95% confidence interval of mean cortical FBP SUVRs relative to the whole cerebellum in a young control group, resulting in 1.11 SUVR or 20 CL^66^.

Structural brain MRI data (3D T1-weighted images) were acquired using 3 T scanners. FreeSurfer version 5.1 was used by the ADNI MRI Core for quantification of regional thickness and volumes based on the 2010 Desikan-Killany atlas^67^. Hippocampal volumes were first adjusted for total intracranial volume (ICV) using a residual modeling method based on data from CU individuals^68^. Normal aging effects on hippocampal volumes were estimated within amyloid-negative CU individuals and regressed out using age and cubic age as predictors^69^. A composite meta-ROI cortical thickness measurement, a previously validated early AD signature^70,71^, was calculated as the surface-area weighted average of the mean cortical thickness in the following individual ROIs: entorhinal, inferior temporal, middle temporal, and fusiform.

### Plasma biomarker measurements

Plasma NfL levels were analyzed at the Clinical Neurochemistry Laboratory, University of Gothenburg, Mölndal, Sweden, using a single molecule array platform (Quanterix, Lexington, MA, USA), as described in detail previously^72^.

### CSF biomarker measurements

CSF was obtained by lumbar puncture and levels of Aβ42, total tau, and phosphorylated tau (p-tau181) were measured using the Elecsys® β-amyloid(1-42), Elecsys Total-Tau, and Elecsys Phospho-Tau (181 P) immunoassays, respectively, on a Cobas e 601 analyzer (Roche Diagnostics International Ltd, Rotkreuz, Switzerland).

### Statistical analysis

Differences in baseline participant characteristics between diagnostic groups were examined using one-way ANOVA for continuous variables and chi-square tests for categorical variables. Across all differential and network-based omics analyses, models were adjusted for shared covariates, including age, sex, education, and analytical batch. Modality-specific covariates included RIN for gene expression and estimated immune cell-type composition (neutrophils, monocytes, NK cells, B cells, CD4+ T cells, and CD8+ T cells) for DNAm. All statistical analyses were performed using R version 4.5.2 (R Core Team, 2025)^73^. Unless otherwise specified, significance for differential and pathway analyses was defined as a BH-FDR < 0.05 to handle multiple comparisons, while a nominal *p* < 0.05 was used elsewhere.

### Differential omics analyses

We performed differential gene expression analysis between AD and CU individuals using the *limma* R package^74^. We fit multivariate linear regression models for each gene with diagnosis as the independent variable, adjusting for shared and gene expression-specific covariates. A contrast matrix was used to compute group differences, and genes were ranked by their moderated t-statistics using empirical Bayes.

Similarly, we identified differentially methylated positions (DMPs) between AD and CU individuals by fitting multivariable linear regression models via *limma* for each CpG site with diagnosis as the independent variable, adjusting for shared and DNAm-specific covariates.

### Network and multi-omics analyses

WGCNA was employed using the *WGCNA* R package to identify modules of highly correlated genes and CpG sites^75^. For both transcriptomic and epigenetic microarray data, we first restricted the dataset to AD and CU individuals, then regressed out the effects of shared and modality-specific covariates. For DNAm, we selected the top 50% most variable CpGs (*n* = 372,153) to focus on sites most likely to reflect biologically meaningful methylation patterns across samples and to reduce computational burden.

Networks were constructed from the adjusted matrices using a signed network type, soft-threshold power of 14, minimum module size of 30 for gene expression or 100 for DNAm, and maximum block size of 10,000. Highly similar modules (correlation >0.75) were merged. The module eigengene (ME), or the first principal component, was calculated to summarize the expression or methylation profile of each module. We fit simple linear regression models via *limma* to identify key modules significantly associated with AD status (*p* < 0.05) for downstream analyses.

Within the key modules, we identified hub genes, which are central to the network’s topology and are often biologically significant. Hub genes were defined as genes with high GS, the p-value from the initial differential analysis with AD status, and high MM, the correlation between the MEs and each gene or CpG site within the module. We selected hub genes that met the criteria of GS < 0.05 and |MM| > 0.8. The network connections among these hub genes were visualized using Cytoscape (v3.10.3)^76^.

MOFA was performed using the *MOFA2* R package to identify latent factors capturing shared variance across both -omics layers in an unsupervised manner^77^. We used matched samples (*n* = 280) from 90 AD and 190 CU individuals, first regressing out the effects of sex, analytical batch, and modality-specific covariates. To balance the dimensionality between datasets and account for the fact that DNAm data is less dynamic in blood compared to gene expression, we included the top 40% most variable genes (*n* = 19,717) and the top 3% most variable CpG sites (*n* = 22,329). We trained the MOFA model with default parameters, except we set the convergence parameter to “slow” and dropped factors that explained less than 2% of the variance. We fit simple linear regression models via *limma* to identify key factors associated with AD status (*p* < 0.05) for further analysis. Within the key factors, we identified hub genes that met the criteria of |weight| > 0.8, scaled per modality.

### Association with AD endophenotypes

We used linear regression models to test cross-sectional associations between the identified -omics signatures of AD (e.g., DEGs, DMPs, modules, MOFA factors) and baseline AD endophenotypes. To assess longitudinal associations, we first estimated individualized rates of change for each endophenotype using simple linear regression and then tested the association between the -omics signatures and these rates. All models were adjusted for age, sex, education, and clinical diagnosis, in addition to modality-specific covariates when using original -omics values. Models for hippocampal volume were not adjusted for age, as the volume measures were already corrected for normal aging.

### Survival analysis for MCI to AD progression

Cox proportional hazards (CPH) regression models were utilized to assess whether the identified -omics signatures of AD (e.g., DEGs, DMPs, modules, factors) could predict MCI-to-AD progression using the *survival* R package^78^. Survival time was defined as the number of years between the baseline MCI diagnosis date to the first AD diagnosis date. If individuals with MCI at baseline did not progress to AD over follow-up, they were right-censored using the number of years between the baseline MCI diagnosis date and last available diagnosis date as their survival time. Individuals that progress to AD and subsequently revert to MCI or CU status were excluded from CPH analysis.

CPH models were adjusted for age, sex, education, and CDR-SB to account for AD severity, in addition to modality-specific covariates when using original -omics values. Identified modules and latent factors were projected onto MCI cases after regressing out the effects of shared and modality-specific covariates, as performed in our original analyses. Specifically, module projection was performed by preserving the original gene/CpG site-module assignments and recalculating the MEs using data from the MCI cases. Prior to latent factor projection, data from MCI cases were additionally scaled to unit variance and centered using the learned intercepts from the trained MOFA model. MCI cases were then projected into the MOFA latent space by fixing the learned factor loadings and approximating factor values using linear least squares regression across both -omics. We validated module and latent factor projection by applying the same projection strategies to the original AD and CU individuals and comparing the reconstructed values to the original modules and factors. Module preservation and correlation analyses showed high concordance between the reconstructed and original values, validating our projection approaches. Significant predictors of MCI-to-AD progression were identified with a nominal *p* < 0.05.

### Functional enrichment and visualization

To interpret the biological meaning of our findings, we performed functional enrichment analysis. GSEA was performed using the *fgsea* R package^79^ to identify pathways enriched among ranked lists of genes (e.g., log2 fold-change, t-statistic, or MM) using the “Hallmark Gene Set Collection” from the Molecular Signatures Database (MSigDB)^80^. For gene co-expression modules, genes were ranked by MM, which allows GSEA to detect pathways driven by hub genes relative to the direction of the module in AD. To maintain visual consistency in functional enrichment bar plots, the direction of the normalized enrichment score (NES) was aligned with the module’s expression direction in AD. For CpG-level data, overrepresentation analysis (ORA) was performed using the *missMethyl* R package, which uses a hypergeometric test that accounts for the varying number of CpG sites per gene^81^. In the case of CpG co-methylation modules, ORA was performed separately on the up- and downregulated CpG sites in AD, as determined by differential methylation analysis, within each module. For both GSEA and ORA, significantly enriched pathways were identified using a BH-FDR < 0.05. To improve interpretation of enrichment results, we organized the 50 hallmark pathways by overarching biological function into 11 categories and ranked them based on their relevance to AD (Supplementary Table S3).

Volcano plots of the DEGs, log-log plots for sex-stratified DEGs, functional enrichment bar plots, and CPH hazard ratio plots were generated using the *ggplot2* R package^82^. The heatmaps of the association results between AD signatures and AD endophenotypes were generated using the *pheatmap* R package^83^. Figures were created in BioRender.

### Sensitivity analyses

Given the influence of major risk factors like sex and *APOE* genotype on AD pathogenesis, we further characterized the molecular signatures in sensitivity analyses. Specifically, we repeated the analyses stratified by sex and after additionally adjusting for *APOE* ε4 carrier status. Additionally, we repeated the differential omics analyses on a subset of 360 participants with amyloid PET-confirmed diagnoses (137 amyloid-negative CU, 160 amyloid-positive MCI, and 63 amyloid-positive AD) to validate that the observed signatures are linked to AD pathophysiology.

## Supplementary information


Supplementary Information
Supplementary Information


## Data Availability

Data used in the preparation of this article were obtained from the ADNI database (adni.loni.usc.edu). As such, the investigators within the ADNI contributed to the design and implementation of ADNI and/or provided data but did not participate in the analysis or writing of this report. A complete listing of ADNI investigators can be found at: http://adni.loni.usc.edu/wp-content/uploads/how_to_apply/ADNI_Acknowledgement_List.pdf.

## Code availability

The code used in this manuscript can be found at https://github.com/cind/adni-mrna-dnam-analysis/.
