# Supplementary material for: Peripheral blood microarray-based transcriptomic and epigenetic analyses identify immune, inflammation, and metabolic dysregulation in Alzheimer’s disease
Source: NPJ Dement. 2026 Aug 31;2(1):74. doi: 10.1038/s44400-026-00100-z (PMC13529590; doi:10.1038/s44400-026-00100-z)
Supplement: Supplementary file 1 [file 44400_2026_100_MOESM1_ESM.pdf]

Characteristic	Overall<br>N = 669	Clinical Diagnosis<br>CU<br>N = 219	MCI<br>N = 347	AD<br>N = 103	p-value
**Age, years**	75 (8)	77 (6)	73 (8)	77 (8)	<0.001
**Women, n (%)**	291 (43%)	109 (50%)	145 (42%)	37 (36%)	0.042
***APOE* ε4 Carrier, n (%)**	264 (39%)	57 (26%)	140 (40%)	67 (65%)	<0.001
**Education Attainment, n (%)**	-	-	-	-	0.123
High School or Less	102 (15%)	22 (10%)	61 (18%)	19 (18%)	-
College	275 (41%)	98 (45%)	135 (39%)	42 (41%)	-
Postgraduate	292 (44%)	99 (45%)	151 (44%)	42 (41%)	-
**RIN**	6.89 (0.63)	6.85 (0.57)	6.92 (0.63)	6.88 (0.72)	0.427
**Amyloid PET-Positive, n (%)**	278 (47%)	55 (29%)	160 (50%)	63 (81%)	<0.001
Missing, n	82	27	30	25	-
**MCI-to-AD Progressor, n (%)**	88 (27%)	-	88 (27%)	-	-
**CDR-SB**	1.67 (2.31)	0.07 (0.29)	1.45 (1.02)	5.84 (2.82)	<0.001
Missing, n	5	2	2	1	-
**MMSE**	27.33 (3.47)	29.08 (1.24)	28.03 (1.69)	21.18 (4.47)	<0.001
Missing, n	6	4	0	2	-
**Change in MMSE/yr**	0.00 (0.69)	0.33 (0.23)	0.08 (0.54)	-1.04 (0.83)	<0.001
Missing, n	12	1	7	4	-
**Amyloid PET, centiloid**	35 (47)	16 (36)	38 (48)	68 (49)	<0.001
Missing, n	95	34	35	26	-
**Change in Amyloid PET, centiloid/yr**	0.06 (2.21)	-0.24 (2.12)	0.12 (2.19)	0.94 (2.44)	0.015
Missing, n	226	56	107	63	-
**CSF p-tau181, pg/mL**	26 (13)	23 (9)	26 (13)	36 (15)	<0.001
Missing, n	197	80	74	43	-
**Change in CSF p-tau181, pg/mL/yr**	0.00 (0.69)	0.02 (0.60)	-0.04 (0.71)	0.08 (0.81)	0.581
Missing, n	335	98	178	59	-
**Plasma NfL, pg/mL**	43 (25)	42 (27)	41 (22)	55 (24)	<0.001
Missing, n	23	7	9	7	-
**Change in Plasma NfL, pg/mL/yr**	0.06 (2.12)	-0.22 (1.68)	-0.09 (2.20)	1.43 (2.32)	<0.001
Missing, n	61	11	24	26	-
**Hippocampal Volume, mm^3**	7,039 (1,054)	7,498 (758)	7,020 (1,032)	5,678 (823)	<0.001
Missing, n	254	101	86	67	-
**Change in Hippocampal Volume, mm^3/yr**	-3 (52)	17 (36)	-4 (52)	-71 (36)	<0.001
Missing, n	271	96	103	72	-
**Meta-ROI Cortical Thickness, mm**	2.74 (0.18)	2.79 (0.13)	2.74 (0.18)	2.48 (0.17)	<0.001
Missing, n	254	101	86	67	-
**Change in Meta-ROI Cortical Thickness, mm/yr**	-0.001 (0.022)	0.006 (0.016)	0.000 (0.022)	-0.029 (0.021)	<0.001
Missing, n	271	96	103	72	-

Table S1a. Demographics for non-Hispanic white participants with available gene expression data and annual trajectories of cognition, neuroimaging measures, and fluid biomarkers. Summary statistics of mean (SD) or n (%) based on complete observations, along with counts of missingness, are shown for study participants by clinical diagnosis with p-values from ANOVA (continuous traits) or chi-square (categorical values) to assess differences between the diagnostic groups. CU, cognitively unimpaired; MCI, mild cognitive impairment; AD, Alzheimer's disease; *APOE* ε4, apolipoprotein E4; RIN, RNA integrity number; PET, positron emission tomography; CDR-SB, clinical dementia rating sum of boxes; MMSE, mini-mental state exam; CSF,

cerebrospinal fluid; p-tau181, phosphorylated tau 181; NfL, neurofilament light chain; meta-ROI, meta-region of interest.

**Characteristic**	**Overall** N = 553	**Clinical Diagnosis** **CU** N = 190	**MCI** N = 273	**AD** N = 90	**p-value**
**Age, years**	75 (8)	77 (6)	73 (8)	77 (8)	<0.001
**Women, n (%)**	245 (44%)	93 (49%)	118 (43%)	34 (38%)	0.188
***APOE* ε4 Carrier, n (%)**	223 (40%)	46 (24%)	116 (42%)	61 (68%)	<0.001
**Education Attainment, n (%)**	-	-	-	-	0.278
High School or Less	77 (14%)	19 (10%)	42 (15%)	16 (18%)	-
College	232 (42%)	87 (46%)	107 (39%)	38 (42%)	-
Postgraduate	244 (44%)	84 (44%)	124 (45%)	36 (40%)	-
**Amyloid PET-positive, n (%)**	231 (47%)	46 (27%)	129 (50%)	56 (86%)	<0.001
Missing, n	62	20	17	25	-
**MCI-to-AD Progressor, n (%)**	79 (30%)	-	79 (30%)	-	-
**CDR-SB**	1.70 (2.33)	0.07 (0.26)	1.44 (0.98)	5.88 (2.62)	<0.001
Missing, n	4	2	2	0	-
**MMSE**	27.24 (3.56)	29.05 (1.24)	28.03 (1.67)	21.03 (4.39)	<0.001
Missing, n	5	4	0	1	-
**Change in MMSE/yr**	-0.03 (0.72)	0.33 (0.24)	0.07 (0.57)	-1.08 (0.81)	<0.001
Missing, n	2	1	0	1	-
**Amyloid PET, centiloid**	34 (46)	15 (36)	37 (46)	73 (45)	<0.001
Missing, n	72	26	20	26	-
**Change in Amyloid PET, centiloid/yr**	0.11 (2.24)	-0.21 (2.17)	0.18 (2.20)	1.00 (2.49)	0.020
Missing, n	151	43	56	52	-
**CSF p-tau181, pg/mL**	26 (13)	23 (9)	26 (13)	37 (15)	<0.001
Missing, n	142	63	41	38	-
**Change in CSF p-tau181, pg/mL/yr**	0.01 (0.69)	0.04 (0.61)	-0.04 (0.70)	0.09 (0.84)	0.516
Missing, n	251	79	123	49	-
**Plasma NfL, pg/mL**	43 (24)	42 (27)	40 (19)	55 (25)	<0.001
Missing, n	19	5	7	7	-
**Change in Plasma NfL, pg/mL/yr**	0.08 (2.12)	-0.17 (1.74)	-0.09 (2.20)	1.33 (2.34)	<0.001
Missing, n	28	5	3	20	-
**Hippocampal Volume, mm^3**	7,054 (1,062)	7,497 (723)	7,052 (1,046)	5,594 (777)	<0.001
Missing, n	198	84	56	58	-
**Change in Hippocampal Volume, mm^3/yr**	-4 (53)	17 (36)	-5 (54)	-76 (31)	<0.001
Missing, n	208	79	68	61	-
**Meta-ROI Cortical Thickness, mm**	2.74 (0.18)	2.79 (0.12)	2.74 (0.18)	2.49 (0.17)	<0.001
Missing, n	198	84	56	58	-
**Change in Meta-ROI Cortical Thickness, mm/yr**	-0.001 (0.023)	0.006 (0.016)	0.000 (0.023)	-0.029 (0.022)	<0.001
Missing, n	208	79	68	61	-

Table S1b. Demographics for non-Hispanic white participants with available DNA methylation data within ±1 year of gene expression profiling and annual trajectories of cognition, neuroimaging measures, and fluid biomarkers. Summary statistics of mean (SD) or n (%) based on complete observations, along with counts of missingness, are shown for study participants by clinical diagnosis with p-values from ANOVA (continuous traits) or chi-square (categorical values) to assess differences between the diagnostic groups.

Transcript	HR [95% CI]	p-value
TMEM132A / 11723445_a_at	1.221 [0.438, 3.398]	0.703
NR4A1 / 11717994_a_at	0.699 [0.499, 0.977]	**0.036**
MXD3 / 11746877_a_at	0.280 [0.084, 0.929]	**0.038**
MAPK14 / 11742204_a_at	1.124 [0.413, 3.065]	0.819
IRAK3 / 11742017_a_at	1.242 [0.652, 2.366]	0.509
GM2A / 11719922_a_at	0.886 [0.495, 1.589]	0.685
CREB5 / 11762865_a_at	1.109 [0.520, 2.361]	0.789
CREB5 / 11722971_a_at	0.901 [0.222, 3.659]	0.884
CD63 / 11757417_x_at	7.922 [0.102, 613.974]	0.351
CD63 / 11744260_a_at	0.472 [0.004, 49.957]	0.752
CD177 / 11730765_at	0.871 [0.677, 1.120]	0.281

Table S2. Cox proportional hazards regression (HR) of the 11 AD-associated differentially expressed genes (DEGs) for predicting MCI to AD progression. Models were adjusted for age, sex, education, and CDR-SB after regressing out the effects of RIN and batch from the original gene expression data. Bold indicates significance at $p < 0.05$.

Category (Rank)	Hallmark Pathways
**1. Immune & Inflammatory processes**	INFLAMMATORY_RESPONSE, TNFA_SIGNALING_VIA_NFKB, IL6_JAK_STAT3_SIGNALING, IL2_STAT5_SIGNALING, INTERFERON_ALPHA_RESPONSE, INTERFERON_GAMMA_RESPONSE, COMPLEMENT, ALLOGRAFT_REJECTION
**2. Proteostasis and ER Stress**	UNFOLDED_PROTEIN_RESPONSE, PROTEIN_SECRETION
**3. Cellular Stress, Apoptosis, & DNA Damage**	REACTIVE_OXYGEN_SPECIES_PATHWAY, APOPTOSIS, HYPOXIA, P53_PATHWAY, DNA REPAIR, UV_RESPONSE_UP, UV_RESPONSE_DN
**4. Mitochondrial Bioenergetics**	OXIDATIVE_PHOSPHORYLATION, GLYCOLYSIS, HEME_METABOLISM
**5. Lipid Metabolism**	FATTY_ACID_METABOLISM, CHOLESTEROL_HOMEOSTASIS, BILE_ACID_METABOLISM, PEROXISOME
**6. Vascular Function & BBB Integrity**	ANGIOGENESIS, COAGULATION, APICAL_JUNCTION, APICAL_SURFACE
**7. Cellular Growth & Proliferation**	E2F_TARGETS, G2M_CHECKPOINT, MYC_TARGETS_V1, MYC_TARGETS_V2, KRAS_SIGNALING_UP, KRAS_SIGNALING_DN, MITOTIC_SPINDLE, MTORC1_SIGNALING, PI3K_AKT_MTOR_SIGNALING
**8. Developmental Signaling**	WNT_BETA_CATENIN_SIGNALING, NOTCH_SIGNALING, HEDGEHOG_SIGNALING, TGF_BETA_SIGNALING, EPITHELIAL_MESENCHYMAL_TRANSITION
**9. Tissue-Specific Differentiation**	SPERMATOGENESIS, ADIPOGENESIS, MYOGENESIS, PANCREAS_BETA_CELL
**10. Hormone Response**	ESTROGEN_RESPONSE_EARLY, ESTROGEN_RESPONSE_LATE, ANDROGEN_RESPONSE
**11. Xenobiotic Metabolism**	XENOBIOTIC_METABOLISM

Table S3. Dictionary of our categorization of the 50 Hallmark pathways into 11 groups based on biological function and ranked by their relevance to AD.

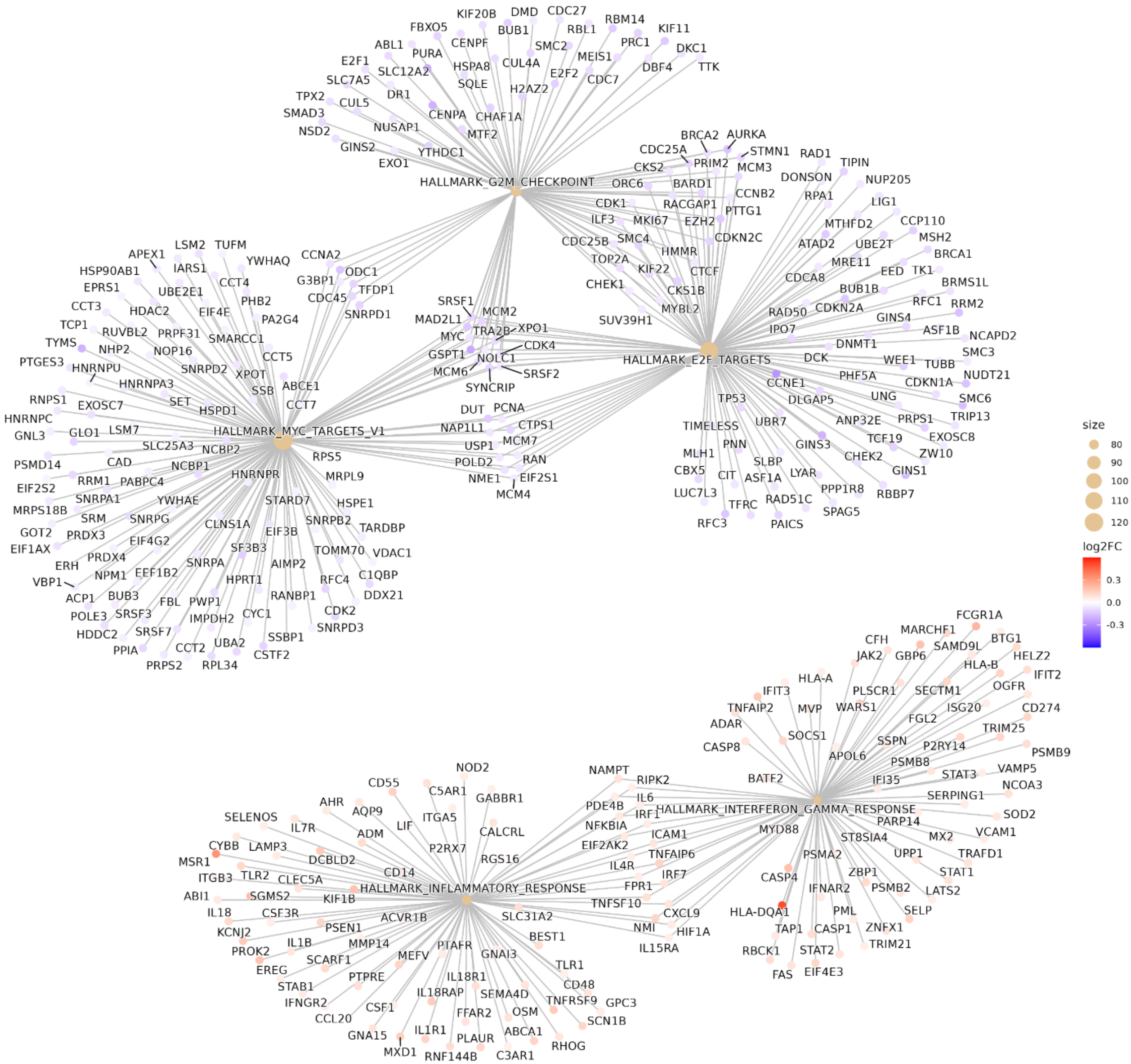

Figure S1. Network plot illustrating the top dysregulated pathways and associated genes in AD. The size of the dot at each pathway name corresponds to the number of associated genes (i.e., the larger the dot the greater number of genes). Red dots represent the pathways with increased enrichment in AD (i.e., interferon gamma response and inflammatory response), while blue dots represent the pathways with decreased enrichment in AD (i.e., MYC targets V1, E2F targets, and G2/M checkpoint).

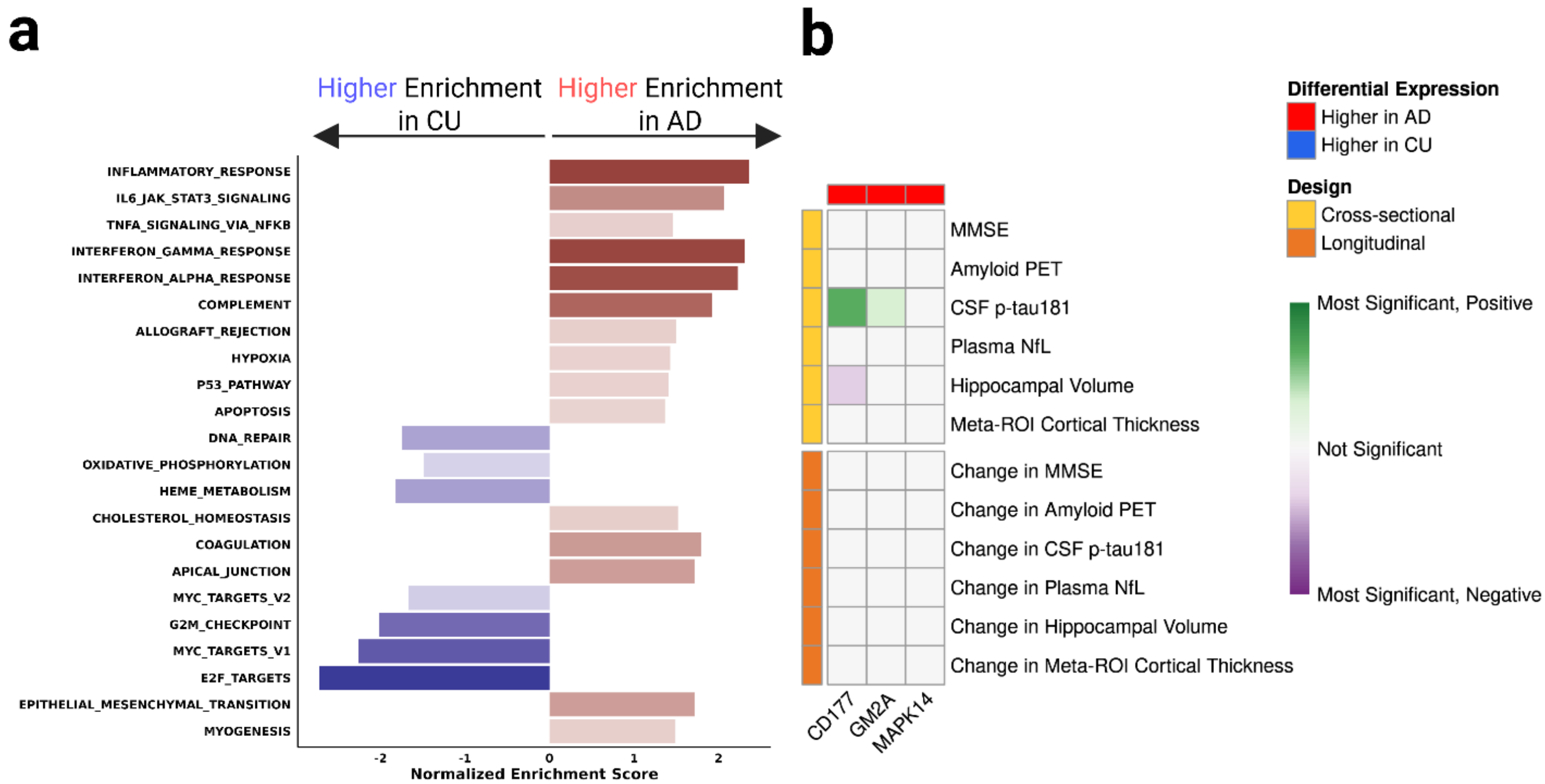

Figure S2. **a**) Bar plot showing the top enriched Hallmark pathways (BH-FDR < 0.05) for *APOE*-independent and AD-associated DEGs. **b**) Heatmap of associations between AD-associated DEGs that were independent of *APOE* ε4 genotype and AD endophenotypes, separated by cross-sectional and longitudinal design.

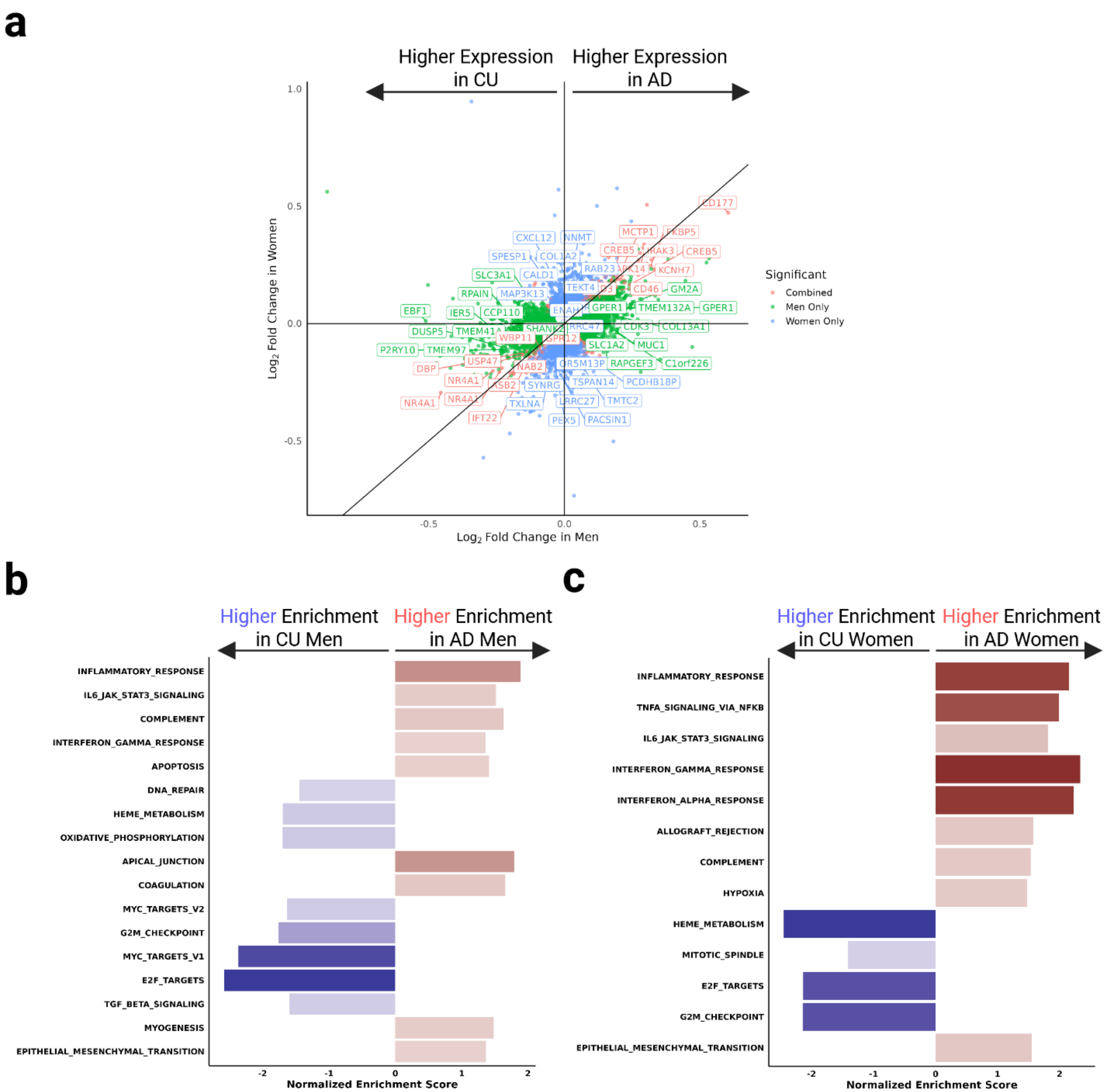

Figure S3. **a**) Log-log plot of AD-related DEGs in men compared to women. Dots correspond to genes with a nominal $p < 0.05$ and labels were only added for the top 10 genes in each sample and direction combination. Red dots represent genes that were identified using the combined sample of men and women, green dots represent genes that were identified in men only, and blue dots represent genes that were identified in women only. **b**) Bar plot showing the top enriched Hallmark pathways (BH-FDR < 0.05) in men. **c**) Bar plot showing the top enriched Hallmark pathways (BH-FDR < 0.05) in women.

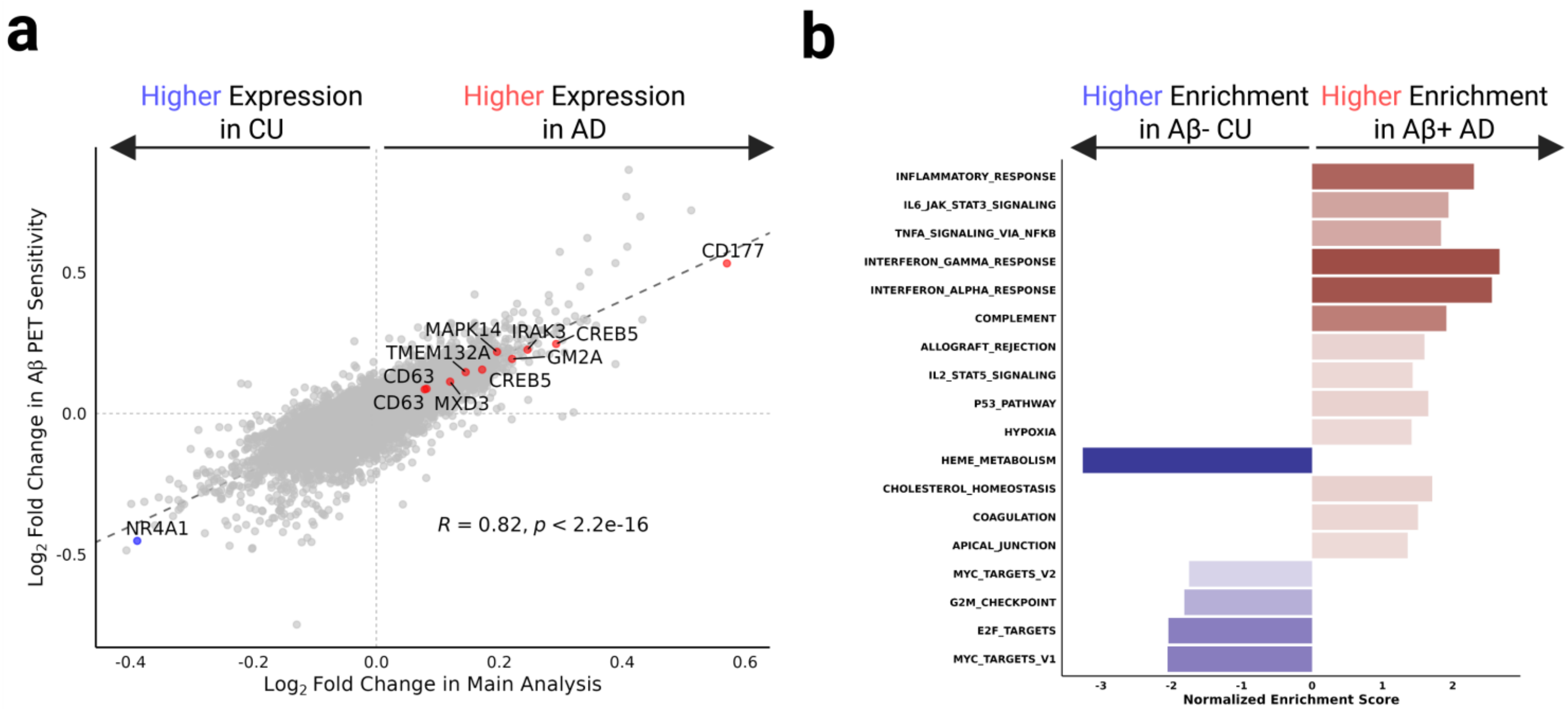

Figure S4. **a**) Log-log plot of the AD-related transcripts in the main analysis compared to the amyloid PET-confirmed diagnosis sensitivity analysis. Dots correspond to the differential expression effect size (log2 fold-change) of all examined genes. Colored dots and labels were only added for the 11 AD-related transcripts that were identified in the main analysis. Red dots represent the 10 transcripts that were upregulated in AD, while the blue dot represents the one transcript that was downregulated in AD. Correlation of the effect sizes between the main and sensitivity analyses was used to assess concordance. **b**) Bar plot showing the top enriched Hallmark pathways (BH-FDR < 0.05) in the amyloid PET-confirmed diagnosis sensitivity analysis.

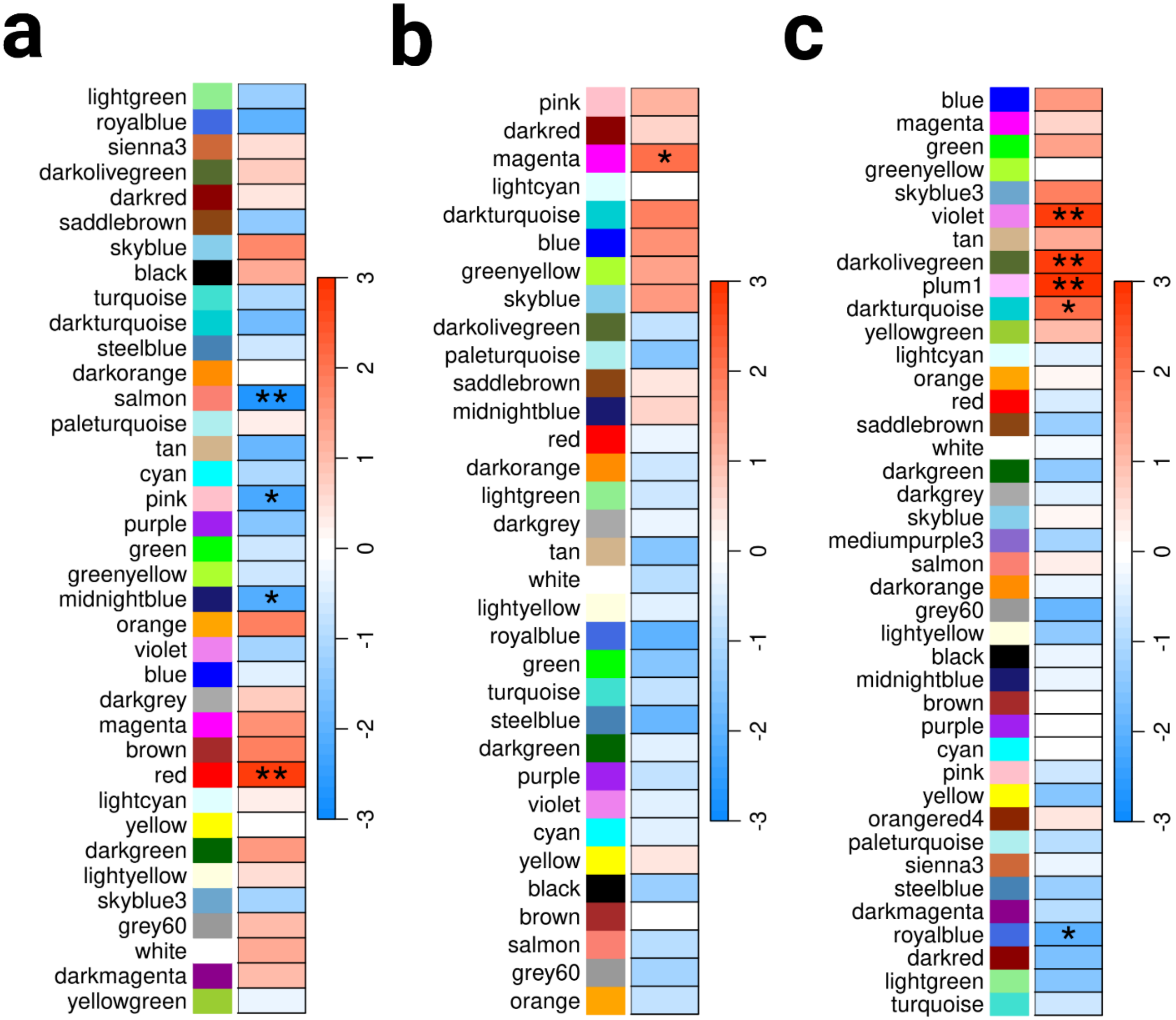

Figure S5. Gene co-expression modules defined using WGCNA and their differential expression in AD patients relative to CU participants in the **a**) overall, **b**) men only, and **c**) women only samples. Red represents upregulated expression in AD, while blue represents downregulated expression in AD. Asterisk denotes the degree of significance (***: p = 0-0.001, **: p = 0.001-0.01, *: p = 0.01-0.05).

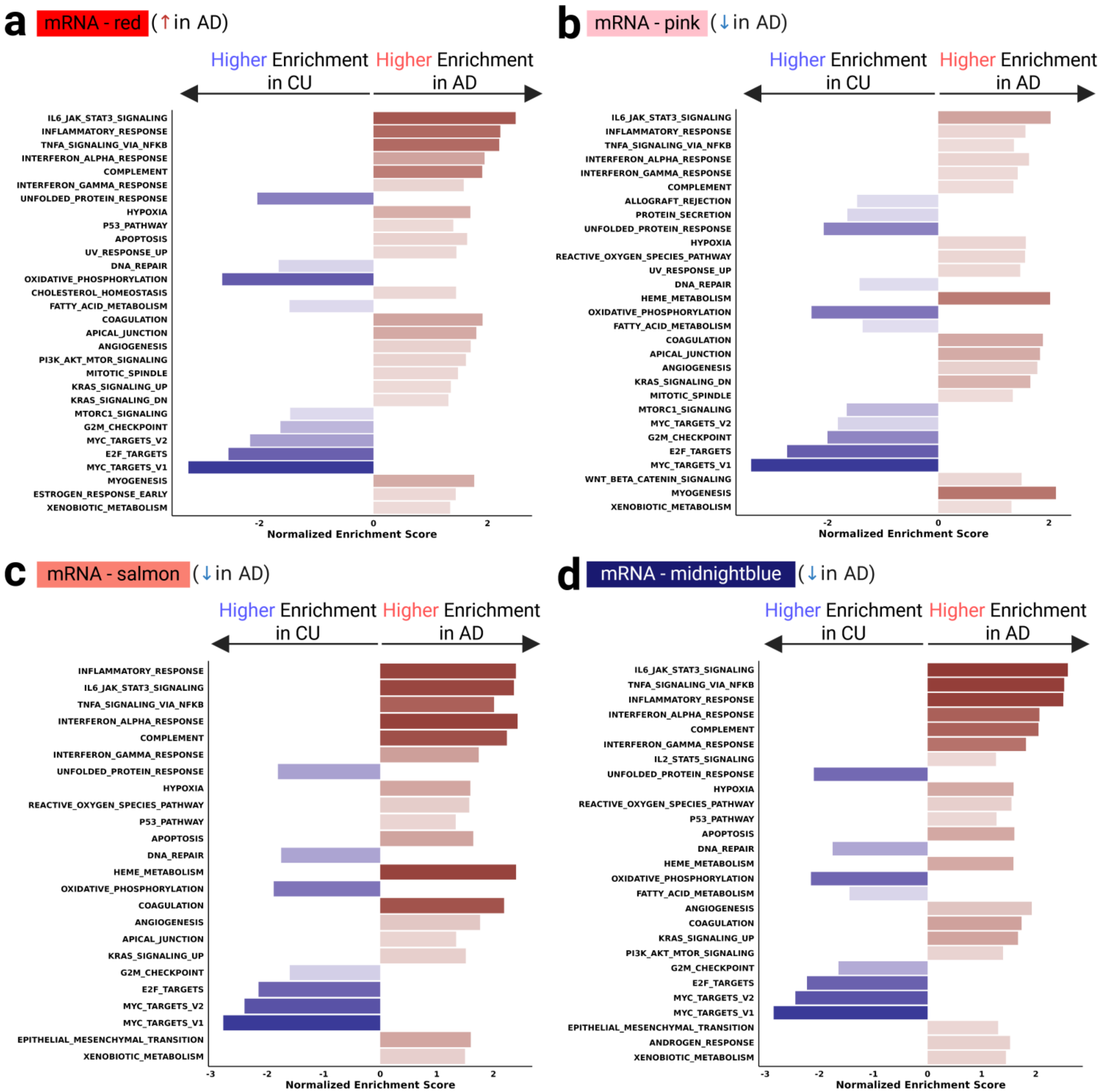

Figure S6. Bar plots showing the top enriched Hallmark pathways (BH-FDR < 0.05) in the **a**) "mRNA - red," **b**) "mRNA - pink," **c**) "mRNA - salmon," and **d**) "mRNA - midnightblue" gene co-expression modules in the overall sample.

a

Sex-stratified gene co-expression networks disrupted in AD						
**Significant in Men**						
**Module**	**Genes**	**Hub Genes**	**Direction in AD**	**Higher Enrichment in AD**	**Higher Enrichment in CU**	**Association with AD Endophenotypes**
mRNA - magenta	685	12	Upregulated*	• Immune & inflammatory processes<br>• Cellular stress & apoptosis<br>• DNA damage<br>• Cholesterol homeostasis<br>• Vascular function & BBB integrity<br>• Developmental signaling<br>• Tissue-specific differentiation<br>• Hormone response	• Proteostasis & ER stress<br>• DNA repair<br>• Mitochondrial bioenergetics<br>• Fatty acid metabolism<br>• Cellular growth & proliferation	• Faster brain amyloid accumulation rate<br>• Slower plasma NfL accumulation rate
**Significant in Women**						
**Module**	**Genes**	**Hub Genes**	**Direction in AD**	**Higher Enrichment in AD**	**Higher Enrichment in CU**	**Association with AD Endophenotypes**
mRNA - royalblue	256	None met the criteria	Downregulated*	• Immune & inflammatory processes<br>• Cellular stress & apoptosis<br>• DNA repair<br>• Mitochondrial bioenergetics<br>• Vascular function & BBB integrity<br>• Tissue-specific differentiation	• Proteostasis & ER stress<br>• DNA damage<br>• Cellular growth & proliferation<br>• Hormone response	• Higher hippocampal volume
mRNA - darkturquoise	187	15	Upregulated*	• Cellular stress<br>• DNA damage<br>• Mitochondrial bioenergetics<br>• Lipid metabolism<br>• Vascular function & BBB integrity	• Immune & inflammatory processes<br>• Cellular growth & proliferation	• Lower MMSE<br>• Faster rate of cognitive decline
mRNA - darkolivegreen	54	3	Upregulated**	• Immune & inflammatory processes<br>• Cellular stress & apoptosis<br>• DNA damage<br>• Cholesterol homeostasis<br>• Vascular function & BBB integrity<br>• Tissue-specific differentiation	• Proteostasis & ER stress<br>• DNA repair<br>• Mitochondrial bioenergetics<br>• Fatty acid metabolism<br>• Cellular growth & proliferation	None met significance threshold
mRNA - violet	54	None met the criteria	Upregulated**	• Immune & inflammatory processes<br>• Cellular stress & apoptosis<br>• DNA repair<br>• Mitochondrial bioenergetics<br>• Vascular function & BBB integrity	• Proteostasis & ER stress<br>• DNA damage<br>• Cellular growth & proliferation	• Slower plasma NfL accumulation rate
mRNA - plum1	34	3	Upregulated**	• Immune & inflammatory processes<br>• Protein secretion<br>• Apoptosis<br>• Developmental signaling<br>• Hormone response	• Unfolded protein response<br>• DNA repair<br>• Mitochondrial bioenergetics<br>• Cellular growth & proliferation	• Higher MMSE

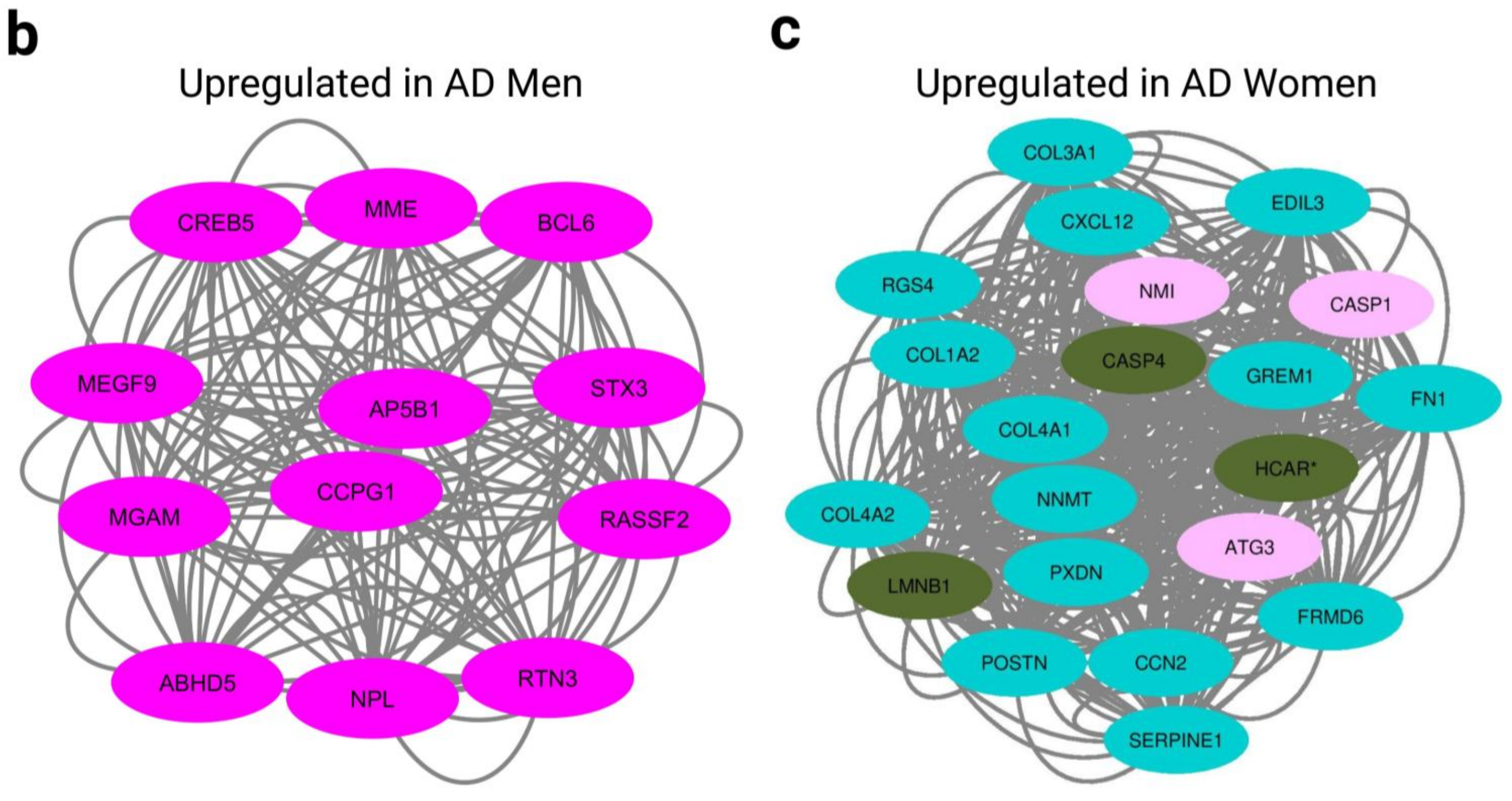

Figure S7. Sex-stratified gene co-expression networks that were dysregulated in AD. **a**) Table showing one gene co-expression network in men and five in women that were significantly dysregulated in AD ($p < 0.05$). The number of genes and hub genes within each network, categories for the top enriched pathways, and

associations with AD endophenotypes are also shown. Asterisk and shading denote the degree of significance (***: p = 0-0.001, **: p = 0.001-0.01, *: p = 0.01-0.05) and the color indicates whether the module was upregulated (red) or downregulated (blue) in AD. **b**) Network plot of the hub genes in the upregulated "mRNA - magenta" module in men. **c**) Network plot of the hub genes in the upregulated modules in women. HCAR* is an abbreviation for *HCAR2* and *HCAR3* which were both mapped to the same probe.

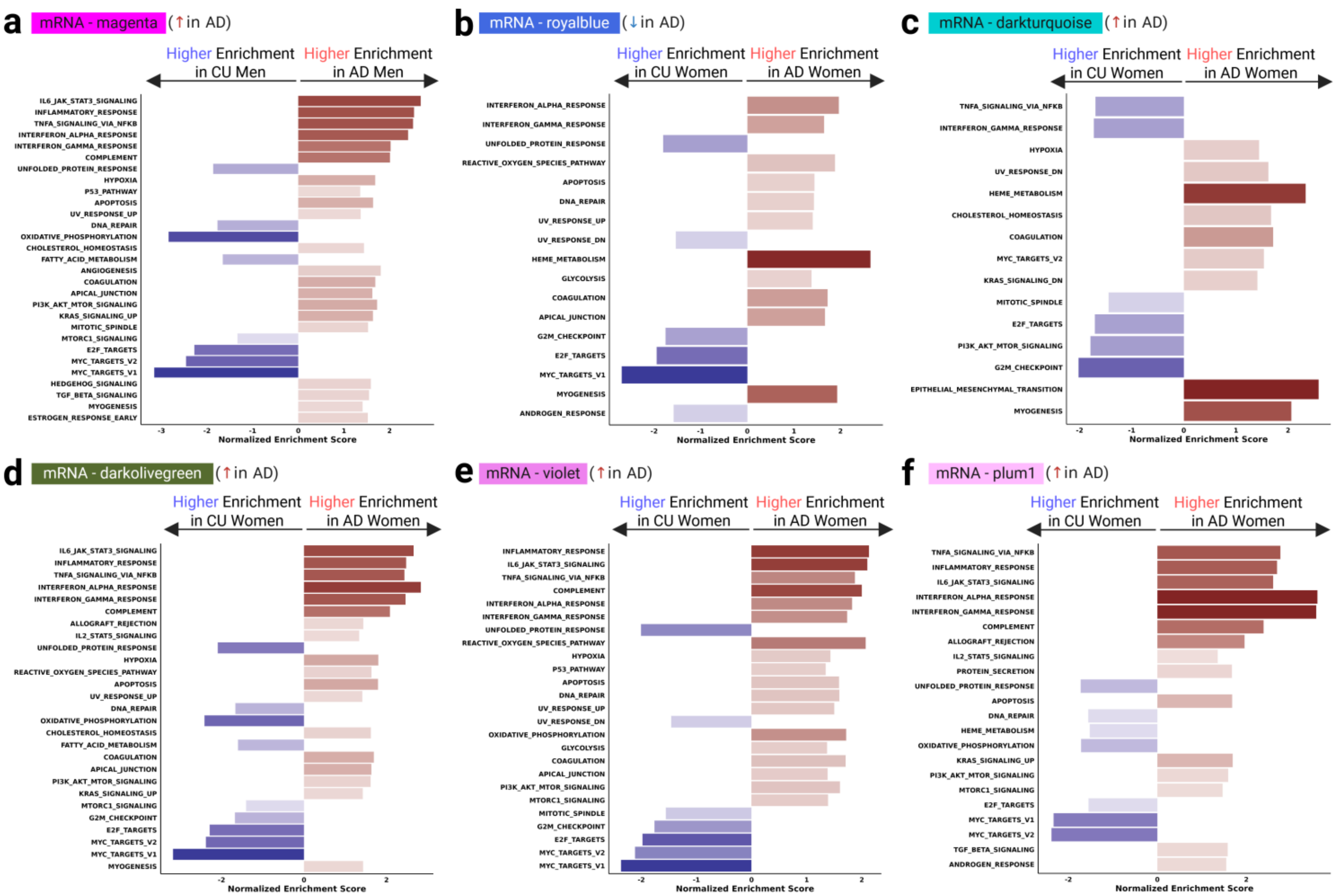

Figure S8. Bar plots showing the top enriched Hallmark pathways (BH-FDR < 0.05) in the **a**) "mRNA - magenta" gene co-expression module in men and the **b**) "mRNA - royalblue," **c**) "mRNA - darkturquoise," **d**) "mRNA - darkolivegreen," **e**) "mRNA - violet," and **f**) "mRNA - plum1" gene co-expression modules in women.

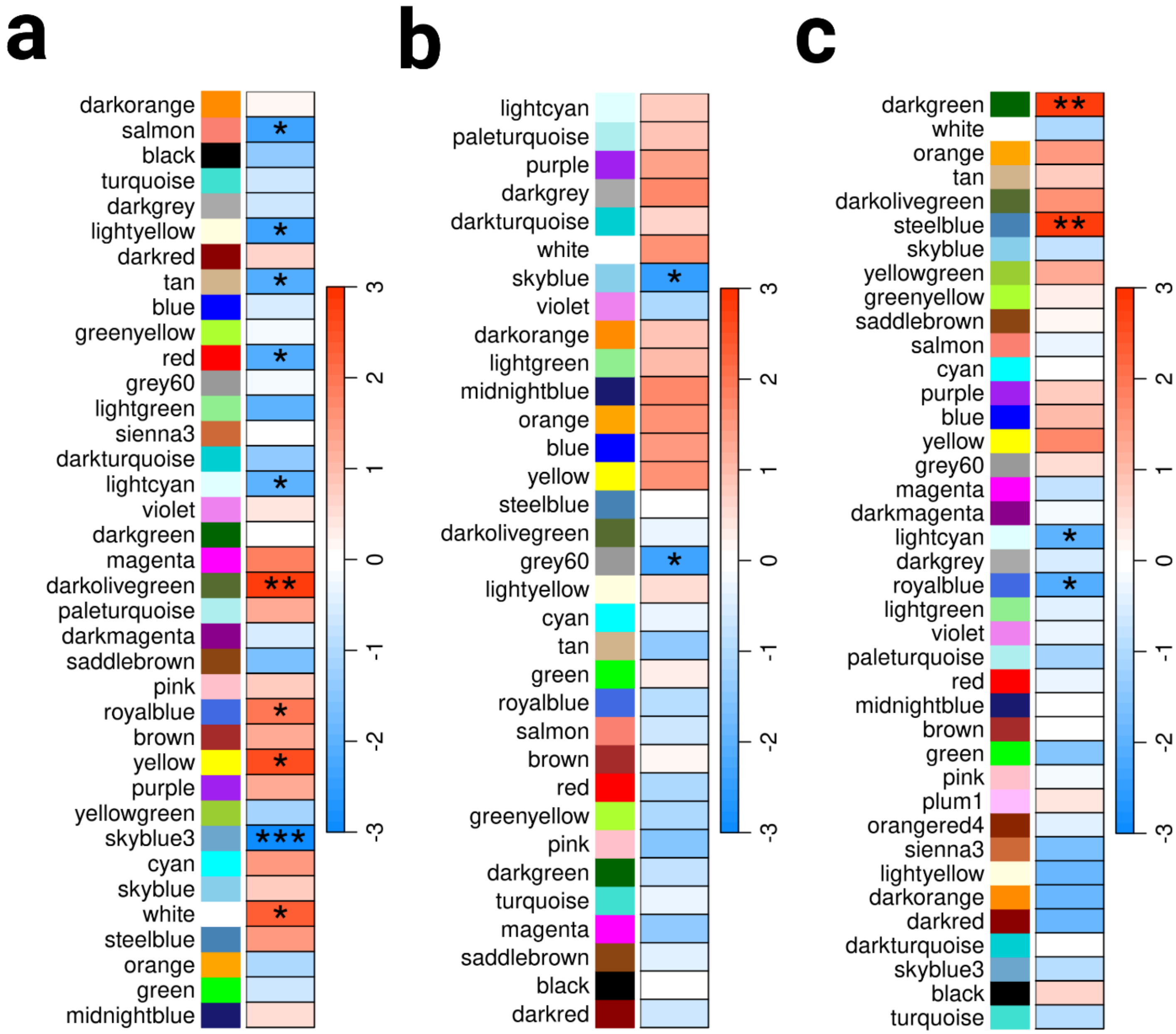

Figure S9. *APOE*-independent gene co-expression modules defined using WGCNA and their differential expression in AD patients relative to CU participants in the **a**) overall, **b**) men only, and **c**) women only samples.

a

Module	Genes	Hub Genes	Direction in AD	Higher Enrichment in AD	Higher Enrichment in CU	Association with AD Endophenotypes
***APOE* -independent gene co-expression networks disrupted in AD**						
**mRNA - yellow**	1958	60	Upregulated*	• Immune & inflammatory processes<br>• Cellular stress & apoptosis<br>• DNA damage<br>• Cholesterol homeostasis<br>• Vascular function & BBB integrity<br>• Tissue-specific differentiation<br>• Hormone response<br>• Xenobiotic metabolism	• Proteostasis & ER stress<br>• DNA repair<br>• Mitochondrial bioenergetics<br>• Fatty acid metabolism<br>• Cellular growth & proliferation	• Faster brain amyloid accumulation rate<br>• Slower plasma NfL accumulation rate
**mRNA - red**	1316	3	Downregulated*	• Immune & inflammatory processes<br>• Cellular stress & apoptosis<br>• Heme metabolism<br>• Vascular function & BBB integrity<br>• Developmental signaling<br>• Hormone response	• Proteostasis & ER stress<br>• DNA repair<br>• Oxidative phosphorylation<br>• Lipid metabolism<br>• Cellular growth & proliferation	None met significance threshold
**mRNA - tan**	388	15	Downregulated*	• Immune & inflammatory processes<br>• Protein secretion<br>• Cellular stress & apoptosis<br>• Heme metabolism<br>• Vascular function & BBB integrity<br>• Developmental signaling<br>• Hormone response<br>• Xenobiotic metabolism	• Unfolded protein response<br>• DNA repair<br>• Oxidative phosphorylation<br>• Cellular growth & proliferation	None met significance threshold
**mRNA - salmon**	378	4	Downregulated*	• Inflammatory processes<br>• Cellular stress<br>• Heme metabolism<br>• Vascular function & BBB integrity<br>• Tissue-specific differentiation	• Immune processes<br>• Proteostasis & ER stress<br>• DNA repair<br>• Oxidative phosphorylation<br>• Lipid metabolism<br>• Cellular growth & proliferation	None met significance threshold
**mRNA - lightcyan**	243	None met the criteria	Downregulated*	• Cellular stress<br>• DNA repair<br>• Mitochondrial bioenergetics<br>• Vascular function & BBB integrity<br>• Tissue-specific differentiation	• Proteostasis & ER stress<br>• DNA damage<br>• Cellular growth & proliferation<br>• Developmental signaling<br>• Hormone response	None met significance threshold
**mRNA - lightyellow**	146	1	Downregulated*	• Cellular stress<br>• Vascular function & BBB integrity<br>• Developmental signaling<br>• Early estrogen response	• Immune & inflammatory processes<br>• Proteostasis & ER stress<br>• DNA repair<br>• Mitochondrial bioenergetics<br>• Lipid metabolism<br>• Cellular growth & proliferation<br>• Tissue-specific differentiation<br>• Late estrogen response	None met significance threshold
**mRNA - royalblue**	114	1	Upregulated*	• Heme metabolism<br>• Vascular function & BBB integrity<br>• Tissue-specific differentiation<br>• Hormone response	• Immune & inflammatory processes<br>• Mitochondrial bioenergetics<br>• Lipid metabolism<br>• Cellular growth & proliferation	• Slower plasma NfL accumulation rate
**mRNA - white**	71	5	Upregulated*	• Cellular stress<br>• DNA damage<br>• Mitochondrial bioenergetics<br>• Lipid metabolism<br>• Vascular function & BBB integrity<br>• Developmental signaling<br>• Tissue-specific differentiation	• Immune & inflammatory processes<br>• Proteostasis & ER stress<br>• Cellular growth & proliferation<br>• Hormone response	• Lower MMSE
**mRNA - darkolivegreen**	36	2	Upregulated**	• Immune & inflammatory processes<br>• Protein secretion<br>• Apoptosis<br>• Oxidative phosphorylation<br>• Hormone response	• Unfolded protein response<br>• DNA repair<br>• Heme metabolism<br>• Cellular growth & proliferation	None met significance threshold
**mRNA - skyblue3**	32	2	Downregulated***	• G2/M checkpoint	• Immune & inflammatory processes<br>• Proteostasis & ER stress<br>• Cellular stress & apoptosis<br>• DNA damage<br>• DNA repair<br>• Mitochondrial bioenergetics<br>• Lipid metabolism<br>• Vascular function & BBB integrity<br>• Cellular growth & proliferation<br>• Tissue-specific differentiation<br>• Xenobiotic metabolism	• Higher CSF p-tau181 levels<br>• Thinner meta-ROI cortex<br>• Faster meta-ROI cortical thinning rate
***APOE* -independent CpG co-methylation networks disrupted in AD**						
**Module**	**CpGs**	**Hub Genes**	**Direction in AD**	**Higher Enrichment in AD**	**Higher Enrichment in CU**	**Association with AD Endophenotypes**
**DNAm - tan**	1946	6	Upregulated*	• Immune & inflammatory processes<br>• Apoptosis<br>• DNA damage	• TNF-α signaling via NF-κB<br>• Allograft rejection	• Thinner meta-ROI cortex<br>• Faster brain amyloid accumulation rate<br>• Slower plasma NfL accumulation rate
**DNAm - lightyellow**	701	12	Upregulated*	None met significance threshold	None met significance threshold	• Higher plasma NfL levels<br>• Thinner meta-ROI cortex<br>• Slower plasma NfL accumulation rate<br>• Faster meta-ROI cortical thinning rate

b

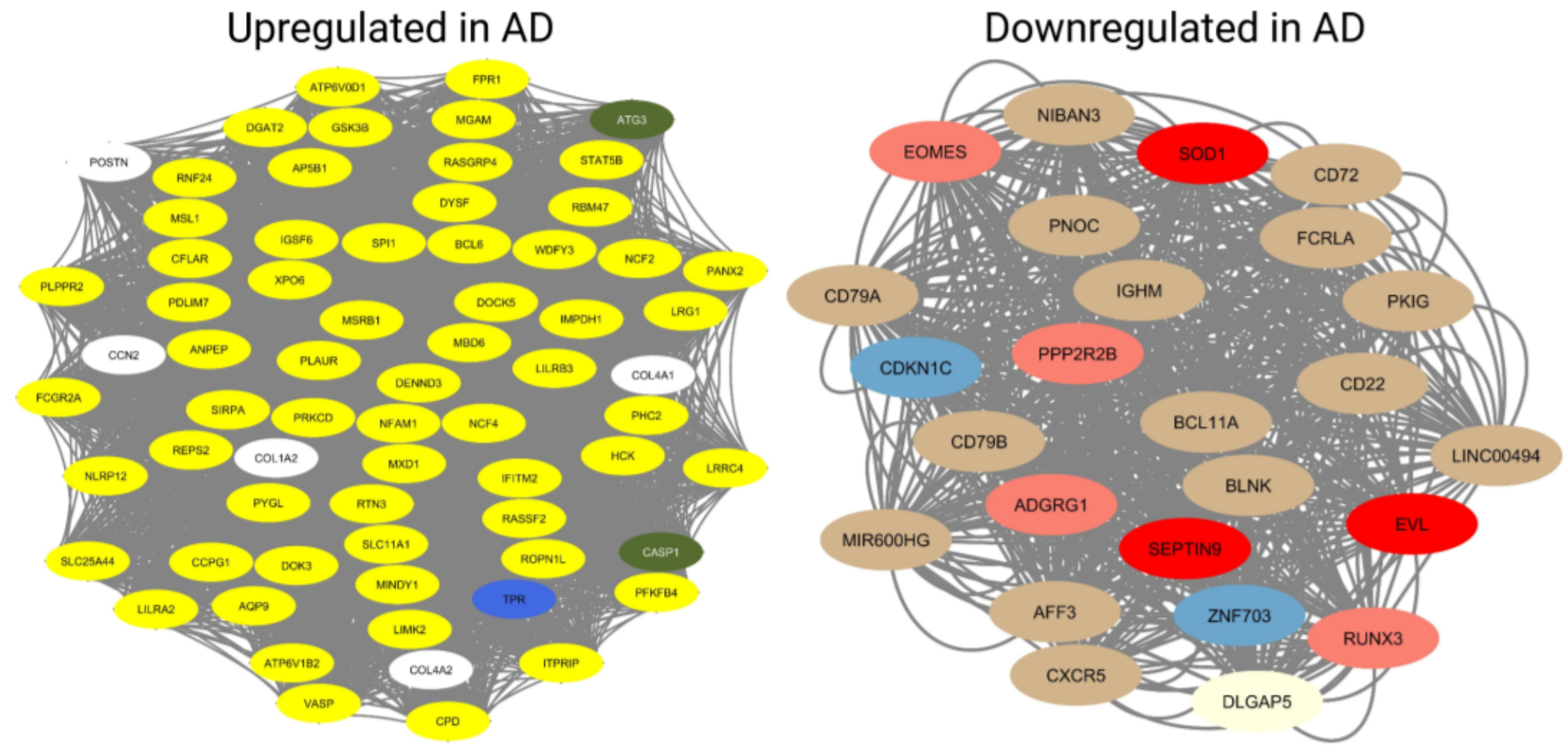

c

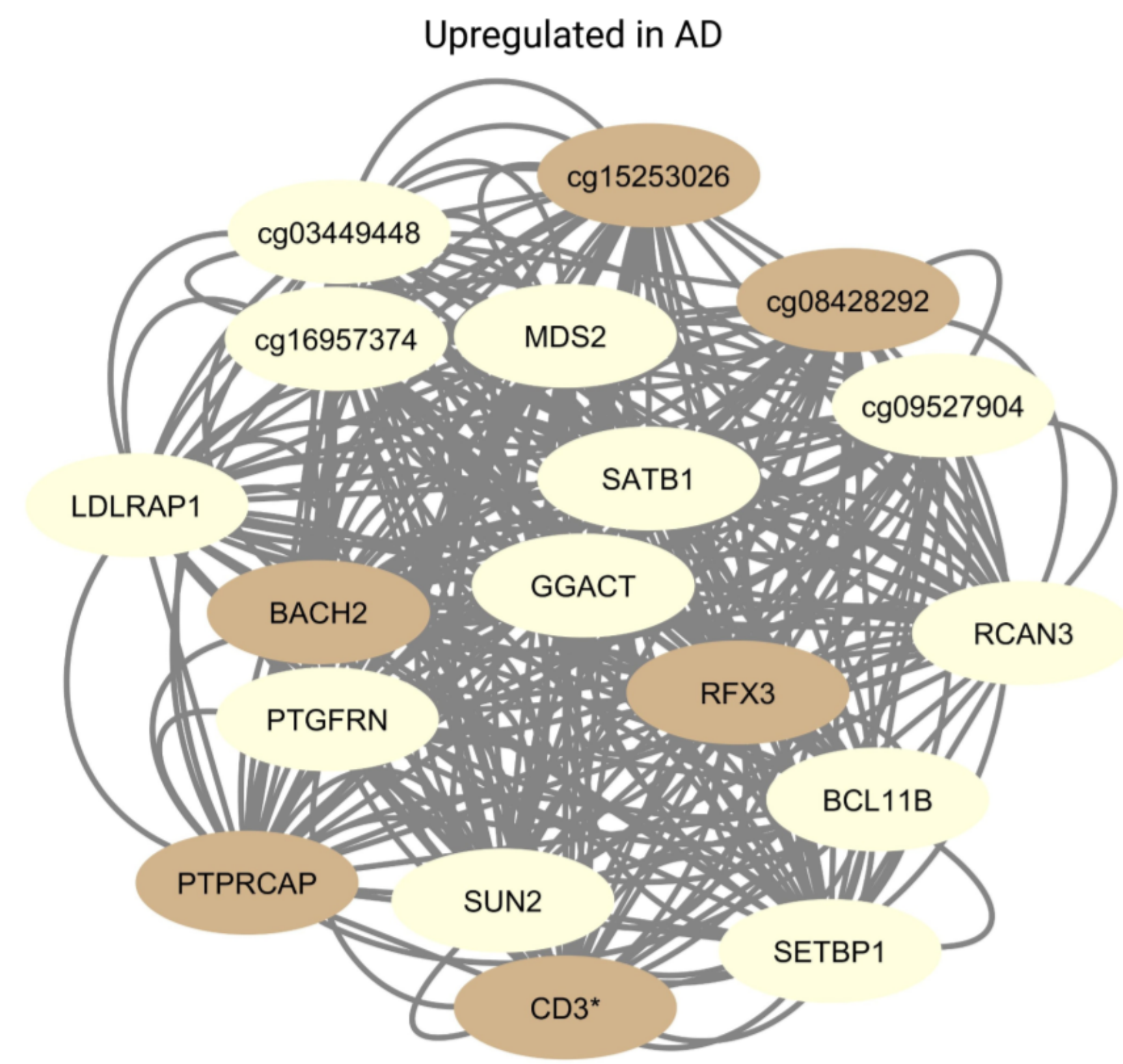

Figure S10. *APOE*-independent gene co-expression and CpG co-methylation networks that were dysregulated in AD. **a**) Table showing 10 gene co-expression networks and two CpG co-methylation networks that were significantly dysregulated in AD ($p < 0.05$). The number of genes, CpG sites, and hub genes within each network, categories for the top enriched pathways, and associations with AD endophenotypes are also shown. **b**) Network plot of the hub genes in the up- and downregulated gene co-expression networks, grouped by direction in AD. **c**) Network plot of the hub genes in the upregulated CpG co-methylation networks in AD. CD3* is an abbreviation for *CD3D* and *CD3G* which were both mapped to the same probe.

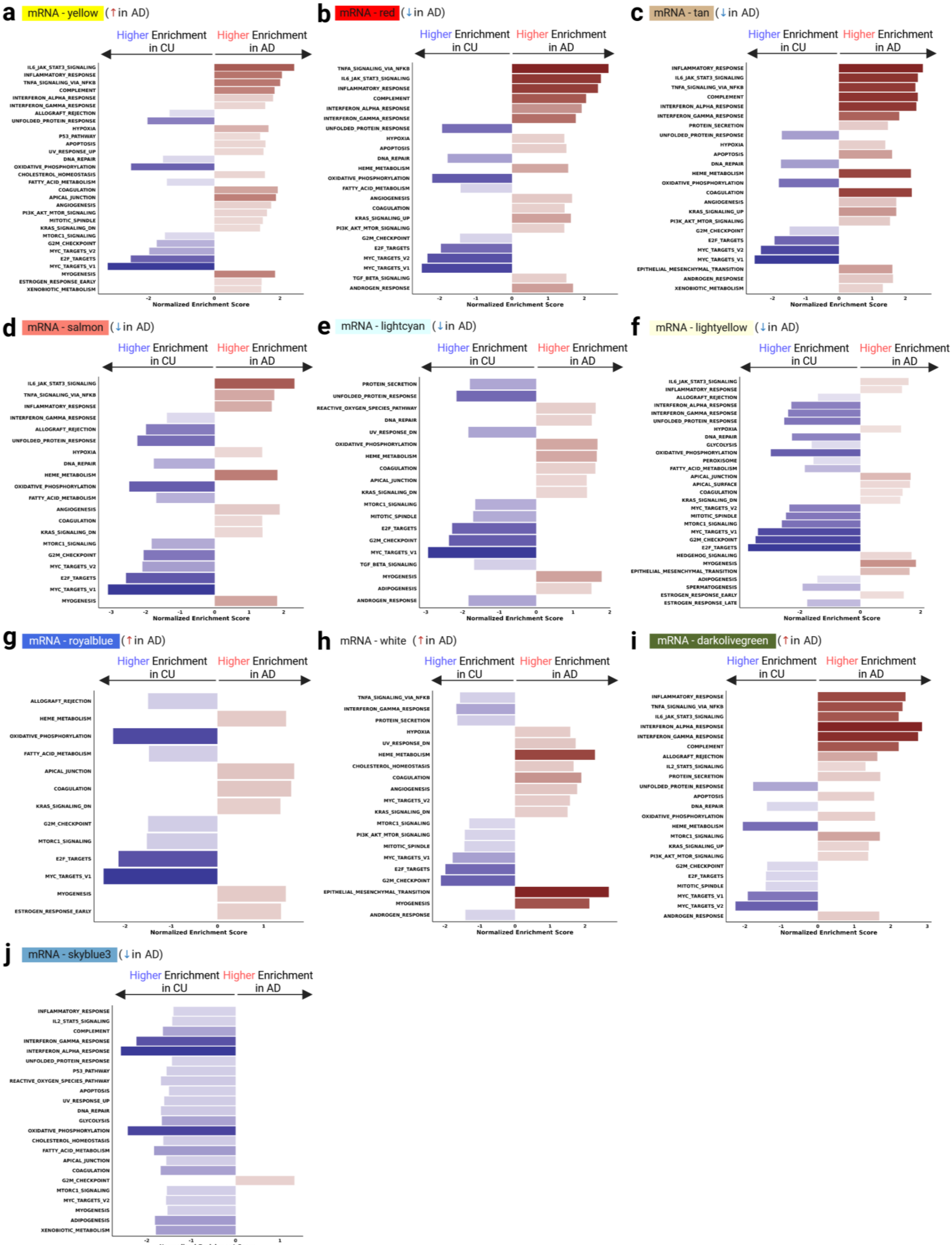
a mRNA - yellow (↑in AD)
Higher Enrichment in CU
Higher Enrichment in AD
Normalized Enrichment Score
b mRNA - red (↓in AD)
Higher Enrichment in CU
Higher Enrichment in AD
Normalized Enrichment Score
c mRNA - tan (↓in AD)
Higher Enrichment in CU
Higher Enrichment in AD
Normalized Enrichment Score
d mRNA - salmon (↓in AD)
Higher Enrichment in CU
Higher Enrichment in AD
Normalized Enrichment Score
e mRNA - lightcyan (↓in AD)
Higher Enrichment in CU
Higher Enrichment in AD
Normalized Enrichment Score
f mRNA - lightyellow (↓in AD)
Higher Enrichment in CU
Higher Enrichment in AD
Normalized Enrichment Score
g mRNA - royalblue (↑in AD)
Higher Enrichment in CU
Higher Enrichment in AD
Normalized Enrichment Score
h mRNA - white (↑in AD)
Higher Enrichment in CU
Higher Enrichment in AD
Normalized Enrichment Score
i mRNA - darkolivegreen (↑in AD)
Higher Enrichment in CU
Higher Enrichment in AD
Normalized Enrichment Score
j mRNA - skyblue3 (↓in AD)
Higher Enrichment in CU
Higher Enrichment in AD
Normalized Enrichment Score

Figure S11. Bar plots showing the top enriched Hallmark pathways (BH-FDR < 0.05) in the *APOE*-independent **a**) "mRNA - yellow," **b**) "mRNA - red," **c**) "mRNA - tan," **d**) "mRNA - salmon," **e**) "mRNA - lightcyan," **f**) "mRNA - lightyellow," **g**) "mRNA - royalblue," **h**) "mRNA - white," **i**) "mRNA - darkolivegreen," and **j**) "mRNA - skyblue3" gene co-expression modules in the overall sample.

a

Sex-stratified, *APOE*-independent gene co-expression networks disrupted in AD						
**Significant in Men**						
**Module**	**Genes**	**Hub Genes**	**Direction in AD**	**Higher Enrichment in AD**	**Higher Enrichment in CU**	**Association with AD Endophenotypes**
**mRNA - grey60**	330	19	Downregulated*	• Immune & inflammatory processes<br>• Cellular stress & apoptosis<br>• DNA damage<br>• Mitochondrial bioenergetics<br>• Lipid metabolism<br>• Vascular function & BBB integrity<br>• Developmental signaling<br>• Tissue-specific differentiation<br>• Xenobiotic metabolism	• Proteostasis & ER stress<br>• DNA repair<br>• Cellular growth & proliferation	• Slower brain amyloid accumulation rate<br>• Slower CSF p-tau181 accumulation rate
**mRNA - skyblue**	59	3	Downregulated*	• Heme metabolism	• Immune & inflammatory processes<br>• Cellular stress<br>• DNA repair<br>• Oxidative phosphorylation<br>• Glycolysis<br>• Lipid metabolism<br>• Vascular function & BBB integrity<br>• Developmental signaling<br>• Tissue-specific differentiation<br>• Hormone response<br>• Xenobiotic metabolism	None met significance threshold
**Significant in Women**						
**Module**	**Genes**	**Hub Genes**	**Direction in AD**	**Higher Enrichment in AD**	**Higher Enrichment in CU**	**Association with AD Endophenotypes**
**mRNA - lightcyan**	300	2	Downregulated*	• Inflammatory processes<br>• Cellular stress<br>• Heme metabolism<br>• Vascular function & BBB integrity<br>• Tissue-specific differentiation<br>• Hormone response	• Immune processes<br>• Proteostasis & ER stress<br>• DNA repair<br>• Oxidative phosphorylation<br>• Lipid metabolism<br>• Cellular growth & proliferation	None met significance threshold
**mRNA - royalblue**	217	5	Downregulated*	• Inflammatory processes<br>• Cellular stress<br>• Vascular function & BBB integrity<br>• Developmental signaling<br>• Myogenesis<br>• Early estrogen response	• Immune processes<br>• Proteostasis & ER stress<br>• DNA repair<br>• Mitochondrial bioenergetics<br>• Lipid metabolism<br>• Cellular growth & proliferation<br>• Spermatogenesis<br>• Late estrogen response	• Lower brain amyloid levels
**mRNA - darkgreen**	164	15	Upregulated**	• Cellular stress<br>• DNA damage<br>• Mitochondrial bioenergetics<br>• Lipid metabolism<br>• Vascular function & BBB integrity<br>• Developmental signaling<br>• Tissue-specific differentiation	• Immune & inflammatory processes<br>• Cellular growth & proliferation	• Lower MMSE<br>• Thicker meta-ROI cortex<br>• Faster rate of cognitive decline
**mRNA - steelblue**	76	1	Upregulated**	• Immune & inflammatory processes<br>• Protein secretion<br>• Apoptosis<br>• Vascular function & BBB integrity<br>• Hormone response	• Unfolded protein response<br>• DNA repair<br>• Mitochondrial bioenergetics<br>• Cellular growth & proliferation	• Higher MMSE

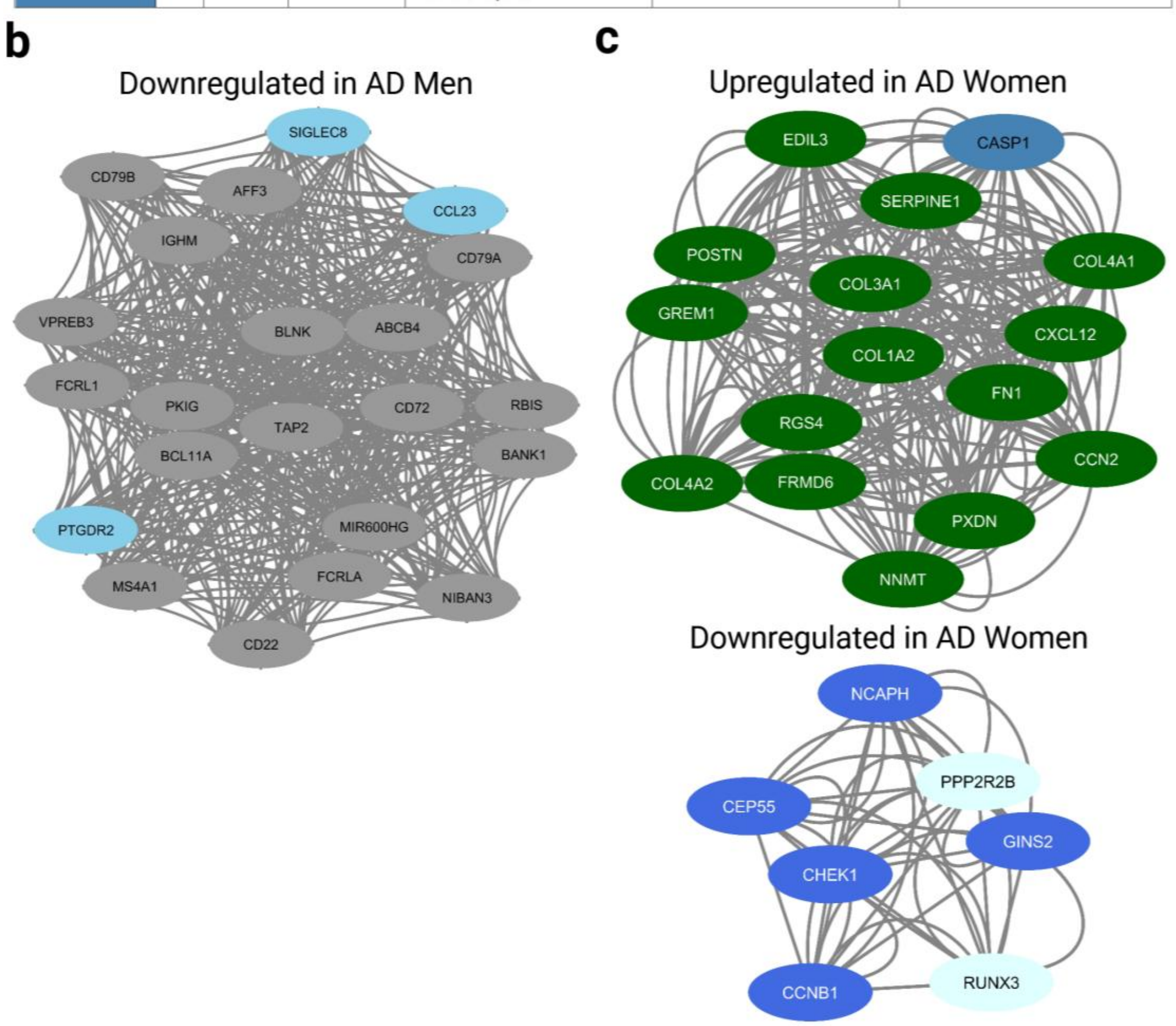

Figure S12. *APOE*-independent and sex-stratified gene co-expression networks that were dysregulated in AD. **a**) Table showing two gene co-expression networks in men and four in women that were significantly dysregulated in AD ($p < 0.05$). The number of genes and hub genes within each network, categories for the top enriched pathways, and associations with AD endophenotypes are also shown. **b**) Network plot of the hub genes in the two downregulated gene co-expression networks in men. **c**) Network plot of the hub genes in the up- and downregulated gene co-expression networks in women, grouped by direction in AD.

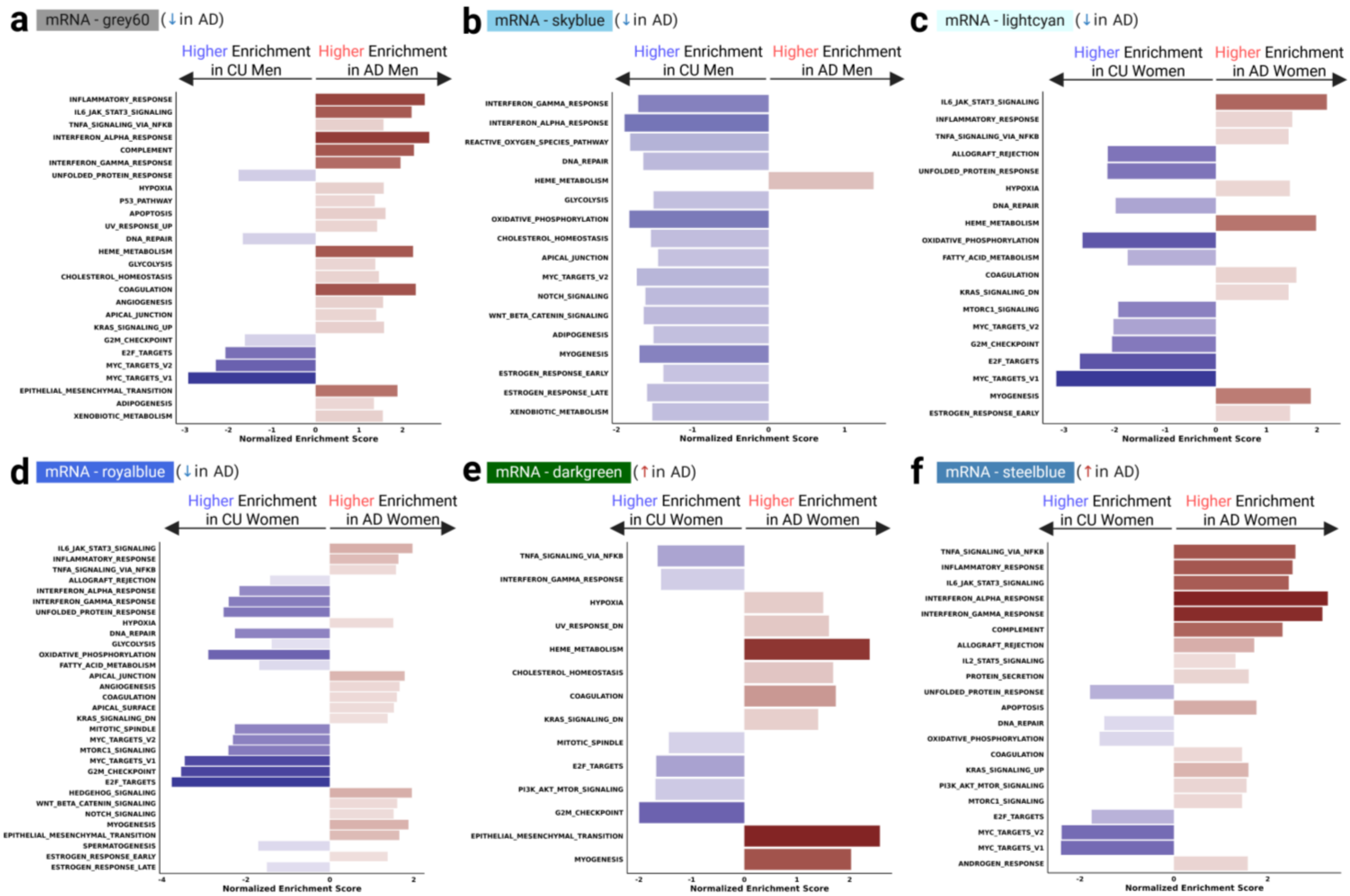

Figure S13. Bar plots showing the top enriched Hallmark pathways (BH-FDR < 0.05) in the *APOE*-independent **a**) "mRNA - grey60" and the **b**) "mRNA - skyblue" gene co-expression modules in men, and the *APOE*-independent **c**) "mRNA - lightcyan," **d**) "mRNA - royalblue," **e**) "mRNA - darkgreen," and **f**) "mRNA - steelblue" gene co-expression modules in women.

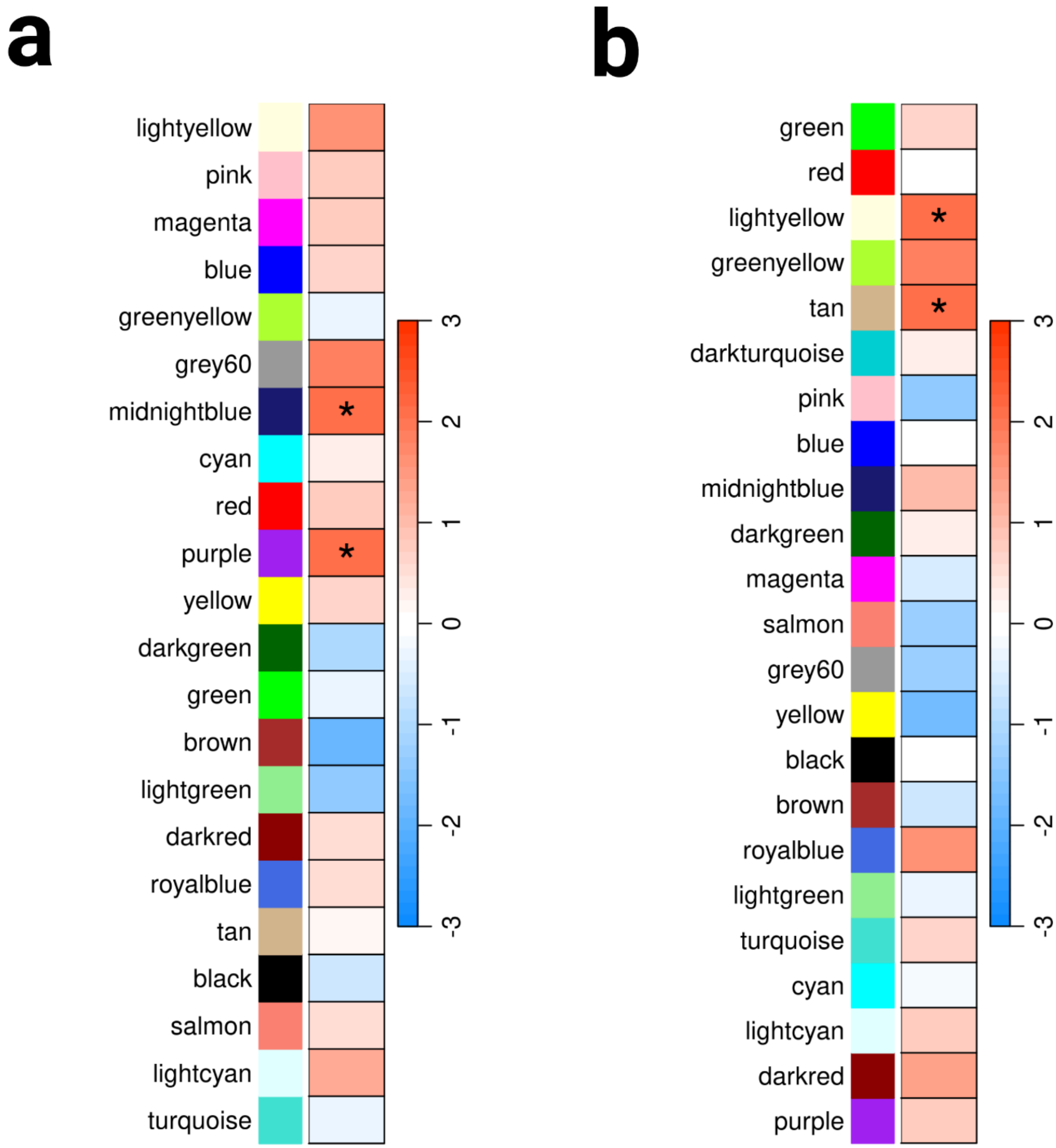

Figure S14. CpG co-methylation modules defined using WGCNA and their differential methylation in AD patients relative to CU participants in the **a**) overall sample and the **b**) *APOE*-independent overall sample.

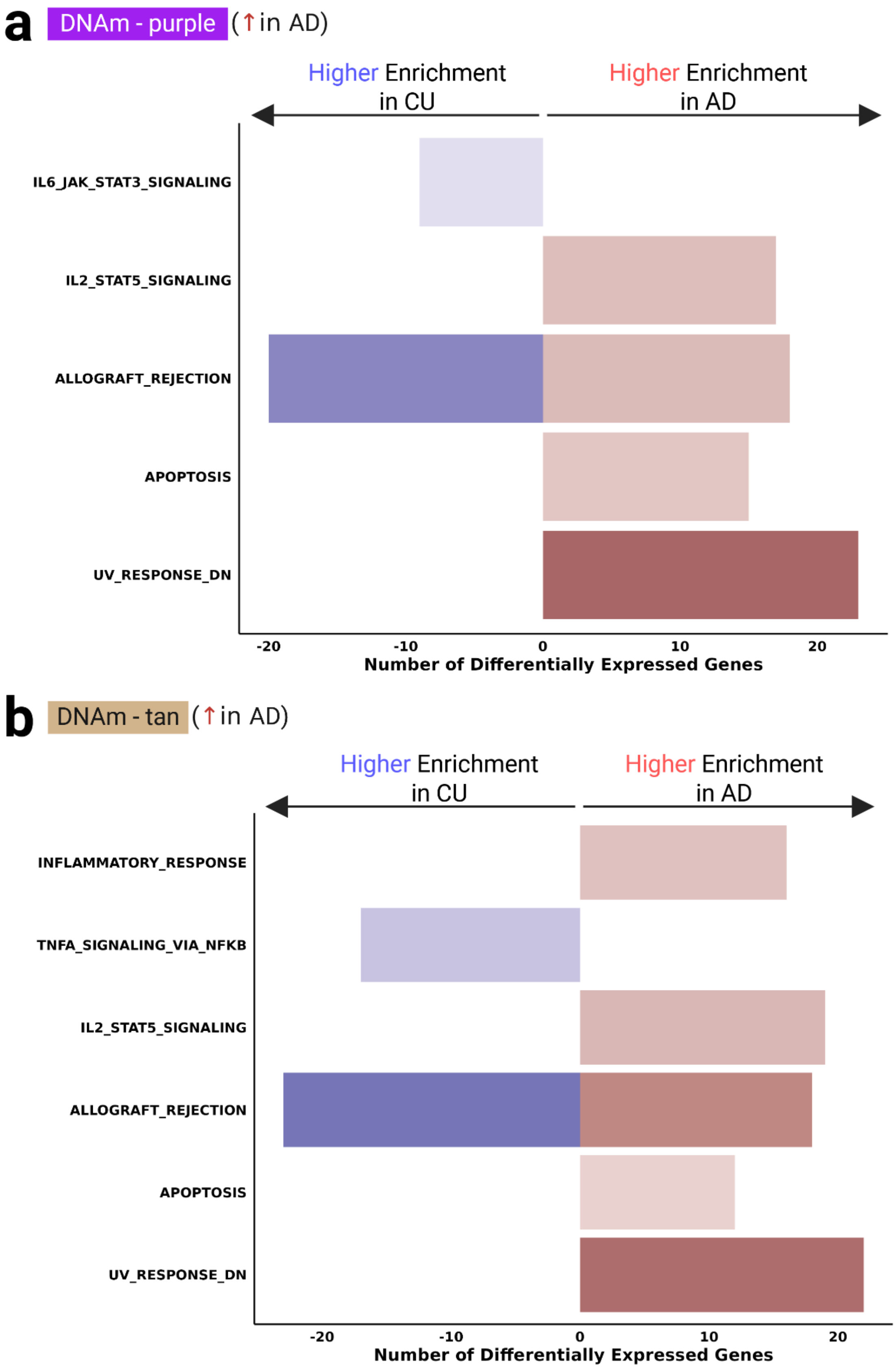

Figure S15. Bar plots showing the top enriched Hallmark pathways (BH-FDR < 0.05) in the **a**) "DNAm - purple" CpG co-methylation module and the **b**) *APOE*-independent "DNAm - tan" CpG co-methylation module.

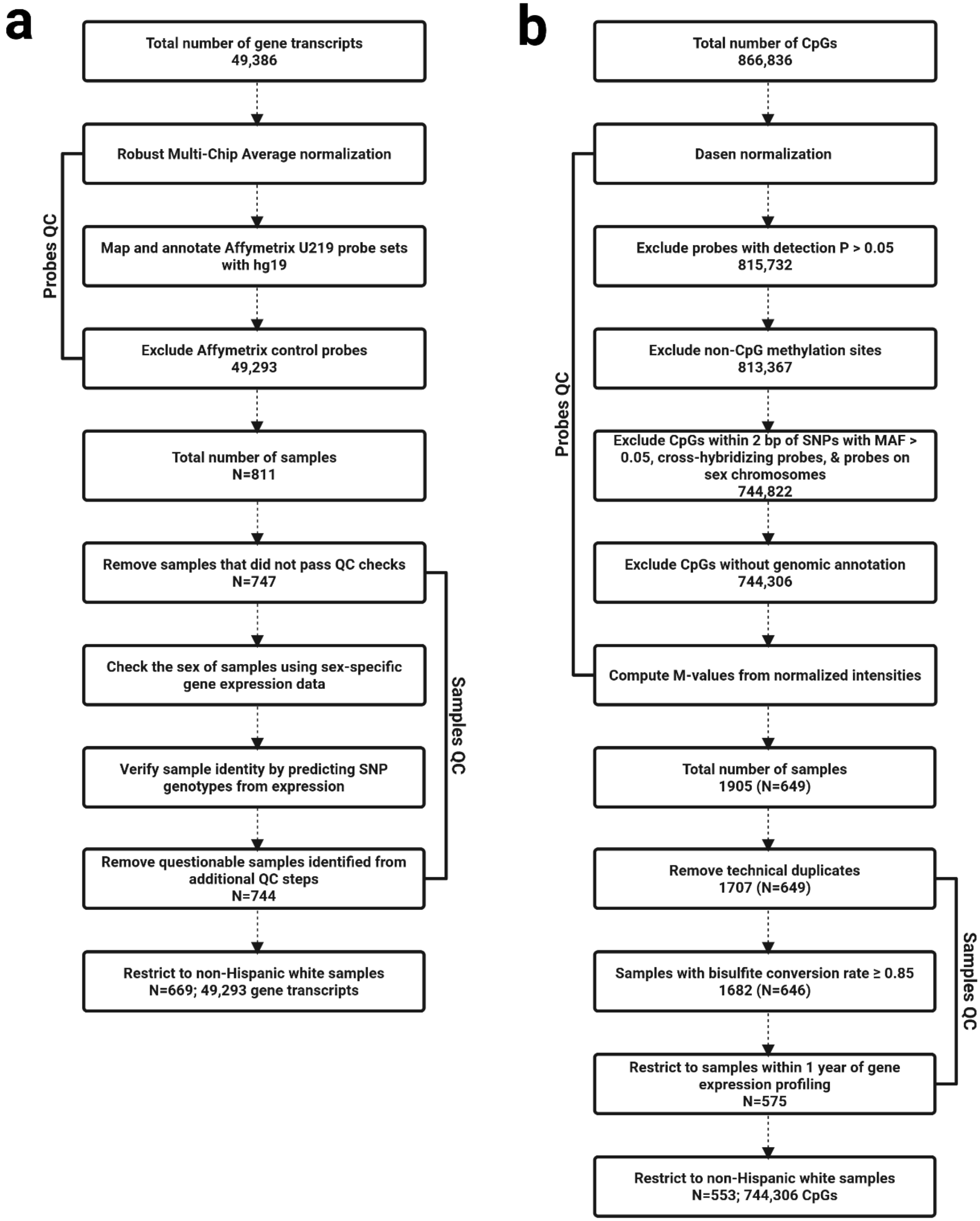

Figure S16. Microarray-based multi-omics preprocessing pipeline. Flowcharts of the preprocessing steps for **a**) gene expression profiling data and for **b**) DNA methylation profiling data. QC, quality control; SNP, single nucleotide polymorphism; bp, base pairs; MAF, minor allele frequency.
